# Cleaved amplified polymorphic sequences (CAPS) marker for identification of two mutant alleles of the rapeseed *BnaA.FAD2* gene

**DOI:** 10.1007/s11033-020-05828-2

**Published:** 2020-09-26

**Authors:** Marcin Matuszczak, Stanisław Spasibionek, Katarzyna Gacek, Iwona Bartkowiak-Broda

**Affiliations:** Research Division in Poznań, Plant Breeding and Acclimatization Institute, National Research Institute, Strzeszyńska 36, Poznań, Poland

**Keywords:** Rapeseed, *Brassica napus*, Mutants, Oleic acid content, CAPS marker, Genotyping

## Abstract

Two mutants of winter rapeseed (*Brassica napus* L. var. *oleifera*) with an increased amount of oleic acid in seeds were created by chemical mutagenesis (HOR3-M10453 and HOR4-M10464). The overall performance of the mutated plants was much lower than that of wild-type cultivars. Multiple rounds of crossing with high-yielding double-low (“00”) cultivars and breeding lines having valuable agronomic traits, followed by selection of high oleic acid genotypes is then needed to obtain new “00” varieties of rapeseed having high oleic acid content in seeds. To perform such selection, the specific codominant cleaved amplified polymorphic sequences (CAPS) marker was used. This marker was designed to detect the presence of two relevant point mutations in the desaturase gene *BnaA.FAD2*, and it was previously described and patented. The specific polymerase chain reaction product (732 bp) was digested using *Fsp*BI restriction enzyme that recognizes the 5′-C↓TAG-3′ sequence which is common to both mutated alleles, thereby yielding band patterns specific for those alleles. The method proposed in the patent was redesigned, adjusted to specific laboratory conditions, and thoroughly tested. Different DNA extraction protocols were tested to optimize the procedure. Two variants of the CAPS method (with and without purification of amplified product) were considered to choose the best option. In addition, the ability of the studied marker to detect heterozygosity in the *BnaA.FAD2* locus was also tested. Finally, we also presented some examples for the use of the new CAPS marker in the marker-assisted selection (MAS) during our breeding programs. The standard CTAB method of DNA extraction and the simplified, two-step (amplification/digestion) procedure for the CAPS marker are recommended. The marker was found to be useful for the detection of two mutated alleles of the studied *BnaA.FAD2* desaturase gene and can potentially assure the breeders of the purity of their HOLL lines. However, it was also shown that it could not detect any other alleles or genes that were revealed to play a role in the regulation of oleic acid level.

**Electronic supplementary material:**

The online version of this article (10.1007/s11033-020-05828-2) contains supplementary material, which is available to authorized users.

## Introduction

Rapeseed (*Brassica napus* L. var. *oleifera*) is an important oilseed crop and is the source of high-quality oil and high-protein animal feed. The current trends in using the rapeseed oil for human nutrition and production of biodiesel have brought new challenges for breeders. For some technologies, especially those used in the food industry, changes in fatty acid profiles of rapeseed oil are needed. The main aim of breeding is to develop new varieties of rapeseed that contain higher amount of oleic acid and lower amount of linolenic acid (called as High-Oleic Low-Linolenic—HOLL varieties) as this oil is considered to be well suited for healthy diet together with better stability and improved frying performance [1–7]. Various methods have been used to develop such varieties, and mutagenesis is the one method that has led to some advance. Two mutants of winter rapeseed (HOR3-M10453 and HOR4-M10464), having increased amount of oleic acid in seeds, were created in the Plant Breeding and Acclimatization Institute, National Research Institute, Research Division in Poznań, by using chemical mutagenesis, with ethyl methanesulphonate (EMS) as the mutagenic factor [8, 9]. The increased oleic acid content in seeds of the original mutants was confirmed by gas chromatography [10, 11], so these mutants can be used as the source of high oleic acid character. However, the overall performance of the mutated plants was much lower than that of wild-type cultivars (compare the mutants with the ‘Monolit’ cultivar in Table S1 in Online Resource 12). To eliminate this disadvantage, some enhancement is needed to obtain new double-low (“00”) varieties of rapeseed having high oleic acid content in seeds. To achieve this goal, mutant genotypes were used for multiple rounds of crossing with high-yielding “00” cultivars and breeding lines having valuable agronomic traits such as yield (including male sterility systems for hybrid breeding), disease resistance, winter hardiness, and other quality traits (e.g. low linolenic acid forms to obtain HOLL varieties). An alternative breeding strategy that may yield some success is the crossing of two different plants with high oleic acid content, of which one is the mutant form, and the second comes from recombinant breeding using natural variability. Using plants with high oleic acid content, which come from two different sources, may increase the stability of the oleic acid level in the resulting offspring and add some elite germplasm to the new segregating lines. In both cases, after each crossing, there are many segregating plants for which the presence or absence of the mutated genes need to be precisely determined. Therefore, to perform the selection of high oleic acid genotypes, it is of great importance for breeders to have some easy-to-use and precise tools to distinguish between mutant and wild-type forms without using the chemical analysis of fatty acid content in seeds. The use of molecular markers for that purpose is a good alternative with an additional advantage, because the analysis is possible in the early seedling stage, thus resulting in the shortened breeding cycle [13]. It is also the only method to distinguish between plants with high oleic acid content of different origin (mutant or natural variability). However, to perform the effective marker-assisted selection (MAS), a reliable method for generating good quality, allele-specific molecular markers must be established. The method must be optimized to obtain precise, repeatable, and explicit results.

**Fig. 1:**
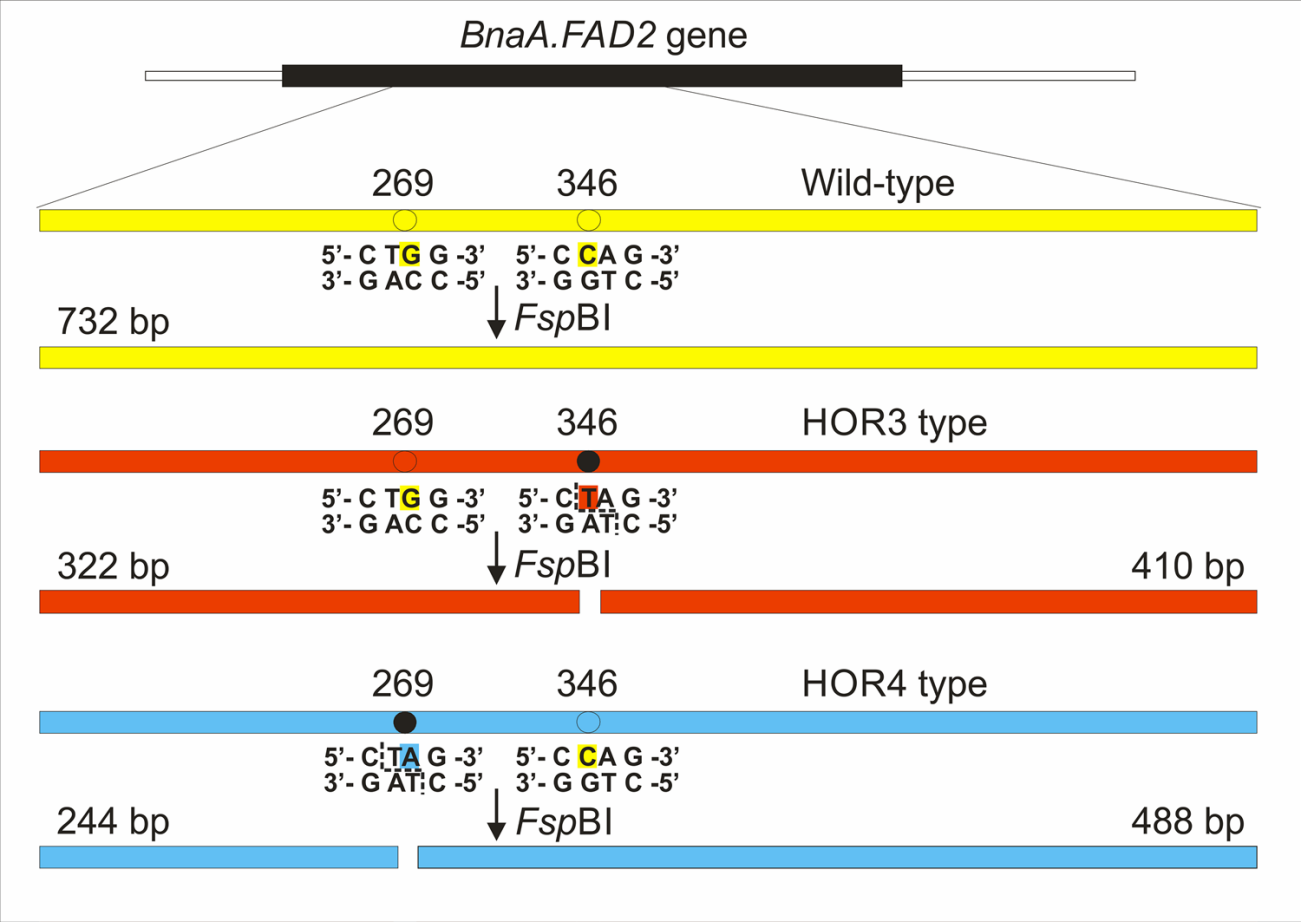
The mutations in the *BnaA.FAD2* gene of rapeseed resulting in the increased amount of oleic acid in seeds and the method to obtain the codominant CAPS marker specific for these mutations (color figure online)

The putative genes responsible for the production of oleic acid in seeds have been identified earlier [14–16]. Among them, the *Bna.FAD2* genes have caught the attention of the research community, as the endoplasmic enzyme coded by these genes (Δ12 oleate desaturase) is known to be the part of the metabolic pathway leading to the conversion of oleic acid into linoleic and linolenic acids. Moreover, the inactivation of such an enzyme may lead to the accumulation of oleic acid in the seeds of rapeseed [14, 17, 18]. Using this information, various methods were used to obtain the modified genotypes and to perform further studies on rapeseed with increased level of high oleic acid. Classical EMS chemical mutagenesis was successfully used by Wells et al. [19] and Bai et al. [20] which is in common with our mutated materials [8, 9]. However, more sophisticated methods, like clustered regularly interspaced short palindromic repeat (CRISPR) / CRISPR-associated nuclease 9 (Cas9)-mediated genome editing [21] or expression of RNA interference (RNAi) constructs, were also used for generation of gene-specific mutations [22, 23] or to perform seed-specific silencing of *Bna.FAD2* genes [24], respectively. To identify and localize the specific mutations in the *Bna.FAD2* genes from our materials, both mutants (HOR3-M10453 and HOR4-M10464) and wild-type forms of rapeseed were studied at INRA, Le Rheu, France [25–27]. The amplification of the putative target genes was performed based on the 1155 bp cDNA sequence (GenBank accession AY577313) [18] (Fig. S1 in Online Resource 1), and the obtained fragments, both from A and C genomes, were cloned and sequenced. The obtained sequences were patented (WO 2007/138,444— [26], US 2009/307,806— [27]). After the comparison of the sequences derived from both mutated and wild-type forms, it was possible to localize the mutations. It was then confirmed that the mutations occurred in the *Bna.FAD2* gene. To date, four copies of *Bna.FAD2* gene were identified in the allotetraploid *B. napus* genome and the authors use different naming conventions to denote them [19, 23, 28, 29]. The genes originating from *Brassica rapa* genome are localized on the A5 and A1 chromosomes and the genes originating from *Brassica oleracea* genome are localized on the C5 and C1 chromosomes. All these genes were found to be transcriptionally active [29], but their expression profiles are different. While the mRNA products coming from the genes on the A5 and C5 chromosomes were present in all tissues, the mRNA products coming from the genes on the A1 and C1 chromosomes were specific for the developing seeds [23, 29]. The observed level of transcript for the A5 copy of the *Bna.FAD2* gene was the highest among all copies (especially during the seed development), and for the A1 and C1 copies this level was very low [23]. Finally, the gene on the chromosome A1 seems to be non-functional due to some deletions and insertions that result in the frameshift during translation and in effect the shortened protein is produced [28, 29]. Thus, the *Bna.FAD2* genes coming from the A5 and C5 chromosomes are considered the main target for the modification of the oleic acid level in seeds of rapeseed. The sequence alignment of studied clones with sequences from *B. rapa* (genome A) and *B. oleracea* (genome C) proved that both mutations are localized in the *BnaA.FAD2* gene of *B. napus* (genome A) [25] (the gene nomenclature follows the rules proposed by Østergaard and King [12]). The previous reports on the non-functionality of the gene coming from the A1 chromosome suggest that the affected gene with the strong effect on the level of oleic acid in seeds must be the one localized on the A5 chromosome—denoted as *BnFAD2-1* [29], *BnaA.FAD2.a* [19, 28] or *BnaFAD2.A5* [23]. The sequence analyses showed that the modified oleic acid content in the two studied mutants is due to the distinct changes in *BnaA.FAD2* gene coding sequence. For HOR3-M10453 mutant, the mutation (C → T) occurred at position 346 of the *BnaA.FAD2* gene. For HOR4-M10464 mutant, the mutation (G → A) occurred at position 269 of that gene (Fig. 1 and Fig. S1 in Online Resource 1). Both mutations resulted in the change of related codon to the STOP codon and in the expression of shorter *BnaA.FAD2* gene product (384 → 115 amino acids for HOR3-M10453 mutant and 384 → 89 amino acids for HOR4-M10464 mutant) [25–27]. The changed protein was no longer functional, and the lack of active enzyme caused the accumulation of oleic acid in the seeds of the mutated plant. Based on the available sequences, the detailed localization of both studied mutations in the *BnaA.FAD2* gene of *B. napus* was compared with the localization of mutations reported by other authors. It was shown that the position of mutations in the studied mutants vary from the position previously reported for the DMS1000 [15] and the SW Hickory [28] mutants (Fig. S1 in Online Resource 1).

The revealed sequences and mutations served as the basis for the design of various molecular markers that can distinguish between two types of mutated and wild-type forms of rapeseed. These markers were theoretically designed based on the obtained sequences, and the respective primer sequences or restriction sites were described in the patent (WO 2007/138,444— [26], US 2009/307,806— [27]). However, these methods had to be redesigned, adjusted to specific laboratory conditions, and thoroughly tested, before implementation and use in MAS. Previously, we described the testing of two polymerase chain reaction (PCR)-based, dominant sequence-characterized amplified regions (SCAR) markers [30]. Each of these markers served to detect different mutations in the *BnaA.FAD2* gene. The results of analyses confirmed that both SCAR markers are valuable tools for detecting the presence of mutated forms and might be used in our breeding programs. The advantage is that the procedure is cheap and easy, as the simple PCR is used for the analysis. However, the disadvantage of this method is that the PCR might be influenced by many factors that may vary between different laboratories. Another issue is that the markers are dominant, and thus, the heterozygous plants could not be distinguished from the homozygous ones. In conclusion, further investigations and studies on codominant markers for the described mutations are needed.

In this study, we present the method for obtaining new, allele-specific, codominant cleaved amplified polymorphic sequences (CAPS) [31] marker that can distinguish between two types of mutated and wild-type alleles. The additional value of this marker is the ability to distinguish between heterozygotes and homozygotes of both types. The marker is based on the sequences described in the patent—similar to the SCAR markers described earlier (WO 2007/138,444— [26], US 2009/307,806— [27]). We adjusted the procedure to better fit it to our laboratory conditions, but the method should also be applicable to other laboratories. We performed several tests using different DNA extraction procedures and technical protocol variants as well as different plant varieties to confirm that the described CAPS marker gives appropriate results for many different genotypes. We also present some examples of the use of this marker for MAS in our breeding programs.

## Materials and methods

### Plant material

The basic set of rapeseed lines for testing the CAPS marker consisted of the following 24 lines of various origins: the original mutant line with the wild-type form of the *BnaA.FAD2* gene—as the negative control sample, sustained by self-pollination (M681 mutant, sample 1 of the basic set) (the mutation in this line refers to the *Bna.FAD3* gene—another desaturase gene that is different from the studied *BnaA.FAD2* gene); the original mutant lines with the mutated *BnaA.FAD2* gene—as positive control samples, sustained by self-pollination (HOR3-M10453 mutant, samples 2 and 3 and HOR4-M10464 mutant, samples 4 and 5 of the basic set) [8, 9]; inbred lines obtained from the crosses between one of the original mutant lines described earlier and one of the selected double-low cultivars (samples 6–11 of the basic set); high oleic acid recombinant plants obtained from the crosses between one of the selected double-low cultivars and the ‘Contact’ cultivar (which is the alternative source of high oleic acid content in winter rapeseed) (samples 12–14 of the basic set); the ‘Monolit’ cultivar—having the typical unchanged fatty acid profile and obtained from ancestors that could not have any mutated allele—as the negative control sample, carrying only the wild-type form of the *BnaA.FAD2* gene (sample 15 of the basic set); and BC_4_ generation plants obtained from the crosses between one of the selected Ogura cytoplasmic male-sterile (CMS) lines and the pollinators being one of the mutant lines described earlier (samples 16–24 of the basic set).

Other plant materials were also used for further testing and validation of the method. It included various varieties and breeding lines derived from the breeding programs, because, besides the testing of the method, many other experiments were performed, including MAS of genotypes with high oleic acid content.

### Preparation of DNA samples

#### The standard DNA extraction procedure (with CTAB)

The DNA was extracted as previously described [32], but with some modifications. The small fragments of leaves of rapeseed were ground to a fine powder in 1.5 ml Eppendorf tubes using liquid nitrogen and autoclavable plastic micropestles. The hot (65 °C) 2×CTAB extraction buffer [2% (w/v) cetyl trimethylammonium bromide (CTAB), 100 mM Tris–HCl (pH 8.0), 20 mM ethylenediaminetetraacetic acid (EDTA), 1.4 M NaCl, 1% (w/v) polyvinylpyrrolidone (PVP), 1% (v/v) β-mercaptoethanol] was then added to the powder. The samples were incubated at 65 °C for 30 min. After mixing with one volume of chloroform-octanol (24:1) (v/v), they were centrifuged at 11,500 × *g* for 10 min. DNA from the collected aqueous phase was precipitated with two-third volume of isopropanol. After 10 min of centrifugation, the supernatant was discarded, and the pellet was resuspended in TE buffer containing 40 μg/ml of RNAse A. After the digestion of RNA contaminants (at 37 °C for 1 h), DNA was again precipitated with isopropanol supplemented with NaCl and finally rinsed with 70% cooled ethanol. The pellet was then resuspended in 100 μl of TE buffer.

#### The automatic FastPrep / CTAB procedure

A major portion of this procedure was based on the standard procedure with CTAB, but the automatic homogenization step was included at the beginning. The small fragments of leaves of rapeseed were placed in 1.5 ml Eppendorf tubes, and then the hot (65 °C) 2 × CTAB extraction buffer [2% (w/v) CTAB, 100 mM Tris–HCl (pH 8.0), 20 mM EDTA, 1.4 M NaCl, 1% (w/v) PVP, 1% (v/v) β-mercaptoethanol] was added. The samples were homogenized automatically using FastPrep® homogenizer (MP Biomedicals, Santa Ana, CA, USA) (40 s, 6 m/s) and the Lysing Matrix A (FastDNA® SPIN Kit, MP Biomedicals). The resulting mixtures were then incubated at 65 °C for 30 min. After mixing with one volume of chloroform-octanol (24:1) (v/v), the samples were centrifuged at 11,500 × *g* for 10 min. DNA from the collected aqueous phase was precipitated with two-third volume of isopropanol. After 10 min of centrifugation, the supernatant was discarded, and the pellet was resuspended in TE buffer containing 40 μg/ml of RNAse A. After the digestion of RNA contaminants (at 37 °C for 1 h), DNA was again precipitated with isopropanol supplemented with NaCl and finally rinsed with 70% cooled ethanol. The pellet was then resuspended in 100 μl of TE buffer.

#### The automatic FastPrep / FastDNA SPIN Kit procedure

The extraction was performed using the FastDNA® SPIN Kit (MP Biomedicals) according to the instruction manual. The small fragments of leaves of rapeseed were placed in 1.5 ml Eppendorf tubes containing a mixture of 800 μl of Cell Lysis Solution (CLS-VF) and 200 μl of Protein Precipitation Solution (PPS) with the Lysing Matrix A (MP Biomedicals). The samples were homogenized automatically using FastPrep® homogenizer (MP Biomedicals, Santa Ana, CA, USA) (40 s, 6 m/s). After 10 min of centrifugation at 11,500 × *g*, the supernatant (700 μl) was transferred to another tube, and an equal volume of Binding Matrix suspension containing guanidine thiocyanate (MP Biomedicals) was added. The mixture was then transferred onto the columns (SPIN Modules containing SPIN Filter, MP Biomedicals) and centrifuged at 11,500 × *g* for 1 min. The suspension on the SPIN Filter was then washed with 500 μl of SEWS-M Wash Solution with ethanol (a mixture of 12 ml of Concentrated SEWS-M Wash Solution and 100 ml of 96% ethanol) (MP Biomedicals) and centrifuged two times. The new SPIN Modules were prepared by moving the SPIN Filters containing the suspension on the new Recovery Tubes. The suspension on the SPIN Filter was then resuspended with 100 μl of the distilled water (MP Biomedicals) and incubated for 5 min at 55 °C. Finally, the columns were centrifuged at 11,500 × *g* for 1 min to recover the samples of pure DNA dissolved in distilled water. No RNAse treatment was used in this procedure.

#### The quick DNA extraction procedure (with SDS)

The DNA was extracted as previously described [33], but with small modifications [34]. The disks of leaf tissue (0.8 cm in diameter) were cut using a cover of a 1.5 ml Eppendorf tube and a piece of filter paper. The tissue fragment was placed in the tube, and the tube was left on ice. The tissue was quickly (ca. 15 s) homogenized using autoclavable plastic micropestles at room temperature, and 400 μl of SDS extraction buffer [0.5% (w/v) sodium dodecyl sulfate (SDS), 200 mM Tris–HCl (pH 9.5), 25 mM EDTA, 250 mM NaCl] was added immediately. The samples were vortexed for 5 s and centrifuged for 1 min. The 300 μl of the resultant supernatant was then mixed with 1 μl of RNAse A (10 mg/ml) to remove RNA contaminants (at 37 °C for 15 min). After the incubation, DNA was precipitated with 300 μl of isopropanol and rinsed with 70% cooled ethanol. Then, the pellet was resuspended in 100 μl of TE buffer.

#### Testing and mixing of DNA samples

The quality and concentration of all DNA samples were tested by separation on a 0.8% agarose gel in Tris–borate-EDTA (TBE) buffer, followed by ethidium bromide staining. The purity and concentration of some samples were additionally analyzed using NanoDrop™ spectrophotometer (Thermo Scientific) for measuring the absorbance (A) at 260, 280, and 230 nm and calculating the A_260_/A_280_ and A_260_/A_230_ ratios. All samples were diluted around 10 times with distilled water (the actual dilution rate depended on the results of concentration tests), and the dilution was followed by molecular marker analyses.

For the heterozygosity detection tests, special mixed DNA samples were prepared. For this purpose, DNAs extracted from two homozygous plants were mixed. Special care was taken to ensure that concentrations of the DNAs extracted from both sources are equal. To achieve this, the samples were mixed after the quality and concentration tests, and the exact volumes and dilution rates of the samples were based on the results of these tests.

### CAPS marker analyses

For the prepared DNA samples, the allele-specific CAPS [31] marker analyses were performed. To obtain the marker, the 732 bp fragment of the *BnaA.FAD2* gene was amplified using two specific PCR primers in the reaction mixture. Both the forward and reverse primers were common to the mutant and wild-type forms; thus, the expected amplified product was equal in all tested plants. The sequences of the primers were as follows: H3H4CON1 forward primer—5′-AGT GTC TCC TCC CTC CAA AAA-3′ and H3H4CON2 reverse primer—5′-ATC GAG GCA ACT CCT TGG A-3′. They were previously designed and patented (WO 2007/138,444— [26], US 2009/307,806— [27]).

The reaction mixture (total reaction volume: 20–40 μl) contained PCR buffer (Fermentas/Thermo Scientific), 1.25 mM of MgCl_2_ (Fermentas/Thermo Scientific), 0.2 mM of each dNTPs (Sigma or Fermentas), 0.6 μM of each specific primers (forward and reverse), 1.2–2.4 U of *Taq* polymerase (Fermentas/Thermo Scientific), and a template DNA (4–8 μl of the diluted sample). Amplification was conducted using an Eppendorf Mastercycler ep Gradient thermal cycler with the following thermal profile: initial denaturation for 4 min at 94 °C (samples were directly transferred from ice into the hot block); 30 cycles for 30 s at 94 °C (denaturation), 30 s at 60 °C (annealing), and 30 s at 72 °C (polymerization); final polymerization for 5 min at 72 °C.

Following the PCR, two different procedures were tested. The basic procedure included purification of the amplified product and the simplified procedure omitted this step. The purification was performed using the GeneJET PCR Purification Kit (Fermentas/Thermo Scientific) according to the instruction manual. Three-fourth of the PCR reaction mixture (30 μl) was taken to another tube, and then an equal volume of Binding Buffer containing guanidine thiocyanate (Fermentas/Thermo Scientific) was added. After the samples were vortexed and centrifuged, the mixture was transferred to the GeneJET Purification Columns (Fermentas/Thermo Scientific) and again centrifuged for 1 min. The samples on the columns were then washed using 700 μl of Wash Buffer with ethanol (mixture of 9 ml of Concentrated Wash Buffer and 45 ml of 96% ethanol) (Fermentas/Thermo Scientific) and centrifuged two times to completely remove any residual Wash Buffer or ethanol. The GeneJET Purification Columns (Fermentas/Thermo Scientific) were then transferred to new 1.5 ml Eppendorf tubes, and 30 μl of the Elution Buffer (10 mM Tris–HCl, pH 8.5) (Fermentas/Thermo Scientific) was added to the center of each GeneJET Purification Column membrane. The columns were centrifuged for a short time to recover ca. 20 μl of purified DNA solution per sample.

The PCR product (purified or not purified) was digested using *Fsp*BI restriction enzyme (Fermentas/Thermo Scientific), which recognizes the 5′-C↓TAG-3′ sequence at the *BnaA.FAD2* gene regions where the mutations occurred. For the digestion reaction, approximately one-third of the initial PCR reaction mixture was used (7–15 μl, depending on the procedure: basic or simplified). Care was taken to maintain an equal amount of DNA in both undigested (control) and digested samples loaded on the agarose gel. The digestion reaction mixture (total reaction volume: 14–30 μl) was prepared by the addition of Tango Buffer (Fermentas/Thermo Scientific) and 10.5–22.4 U of *Fsp*BI restriction enzyme (Fermentas/Thermo Scientific). The enzyme was added as the last component, separately to each sample to avoid its deactivation.

Samples containing undigested or digested PCR products were analyzed by separation on 1.8% agarose gel in the TBE buffer, followed by ethidium bromide staining. The undigested (control) and digested samples from the same plant were always placed side by side on the agarose gel for better comparison. Only approximately one-third of the initial PCR mixture (undigested or digested) was added to each well of the agarose gel (after mixing with an appropriate amount of the Loading Dye Solution—MBI Fermentas). Lambda DNA digested with *Hin*dIII and *Eco*RI restriction enzymes (Fermentas) (250 ng of digested DNA per well) was used as the size marker. The gels were photographed under the UV light using a Vilber Lourmat Quantum ST4 1000 gel imaging system.

### Biochemical analyses (oil content, total glucosinolates content, and fatty acid composition)

The oil content in seeds was determined by the nuclear magnetic resonance (NMR) method using the pulse NMR analyzer (Newport Instruments Ltd). The method was calibrated based on the Soxhlet wet analysis of oil content in rapeseed [35].

Fatty acids from seeds were extracted using hexane, and methyl esters of the extracted fatty acids were obtained. The composition of fatty acids was determined using gas chromatography (Agilent Technologies). The results were calculated as the percentage of each fatty acid compared to the sum of all fatty acids [10, 11].

The glucosinolates from seeds were extracted using methanol with barium acetate. Then, the silyl derivatives of desulfoglucosinolates were obtained, and the analysis of total glucosinolate content (expressed in μmol/g of seeds) was performed by gas chromatography (using instruments from Agilent Technologies) [36].

## Results

The overview of the designed CAPS marker based on the model of the *BnaA.FAD2* gene obtained using the sequence data is presented in Fig. 1. Six basic band profiles can be obtained and visualized after the digestion reaction (Table 1), and the most frequent five profiles obtained experimentally are shown in Fig. 3. These profiles depend on the alleles of the *BnaA.FAD2* gene that are present in the studied plant.

**Table 1:** Band patterns that can be obtained using the universal, codominant CAPS marker for the detection of HOR3 and HOR4 type mutations in the *BnaA.FAD2* gene of rapeseed

No	HOR3 type mutation	HOR4 type mutation	DNA fragments (pattern)
1	Wild-type homozygote	Wild-type homozygote	732 bp
2	Mutated homozygote	Wild-type homozygote	410 bp, 322 bp
3	Heterozygote	Wild-type homozygote	732 bp, 410 bp, 322 bp
4	Wild-type homozygote	Mutated homozygote	488 bp, 244 bp
5	Wild-type homozygote	Heterozygote	732 bp, 488 bp, 244 bp
6	Heterozygote^1^	Heterozygote^1^	488 bp, 410 bp, 322 bp, 244 bp

^1^HOR3 and HOR4 type mutations are in the repulsion phase

Profiles 1–5 were obtained experimentally and are shown in Fig. 3

**Fig. 2:**
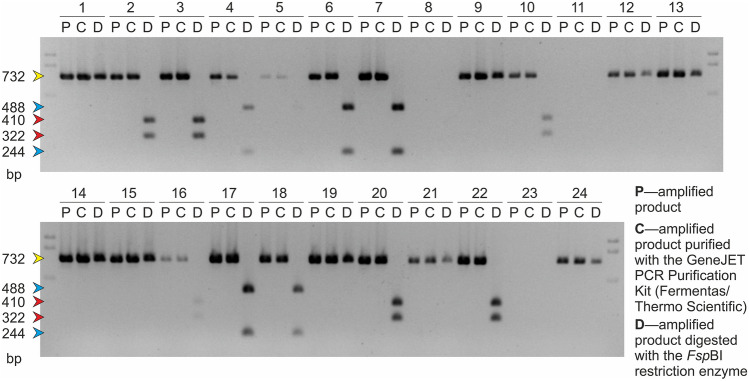
The results of the analyses performed using the CAPS marker specific for the mutations in the *BnaA.FAD2* gene of rapeseed for the basic set of 24 rapeseed lines (see Materials and Methods for the description of each line). The arrows indicate the DNA fragments observed on the agarose gel, and their colors correspond to the colors used for the display of each mutation shown in Fig. 1. For each analyzed plant, three samples (designated with P, C, and D letters, which are explained in the figure) representing three steps of the CAPS protocol were applied on the gel (color figure online)

The performance of the presented marker was tested on the basic set of 24 rapeseed lines that has served previously as the reference for the standardization of two dominant SCAR markers for the same mutations [30] (Fig. S2 in Online Resource 2). These analyses were performed using the standard DNA extraction procedure (with CTAB) and the original CAPS marker protocol that included purification of the amplified product. The results of these analyses are presented in Fig. 2 (compare with Fig. S2 in Online Resource 2).

The repeatability of the marker analysis was checked with the different methods of DNA extraction used (automatic FastPrep / CTAB, automatic FastPrep / FastDNA SPIN Kit, standard with CTAB, and quick with SDS). The analysis of the quality and concentration of the DNA samples indicated some differences between these methods. When using the agarose gel (data not shown), the best quality was observed for the standard method (with CTAB), and the two automatic procedures provide slightly worse results (showing higher level of DNA degradation). The high concentration of DNA in the samples obtained using the standard method was confirmed using NanoDrop™ spectrophotometer. The spectrophotometric analysis of DNA concentration for the remaining samples showed generally much lower values. The good quality of almost all samples was indicated by the correct A_260_/A_280_ ratio that ranged between 1.79 and 1.95. Only a few samples did not fulfill this criterion. Most of the samples exhibiting a low A_260_/A_280_ ratio were prepared using the quick method (with SDS). For this procedure, the obtained concentration of DNA was very low, and so, the bands were hardly visible on the gel (data not shown). This was also confirmed by spectrophotometric analysis. The purity of some samples obtained using this method was also poor, showing the low A_260_/A_230_ ratio of 0.4–1.9. However, it is worth noting that among all the studied samples, the lowest A_260_/A_230_ ratio of approximately 0.1 was observed for the group of DNA samples obtained using the automatic FastPrep / FastDNA SPIN Kit procedure.

Despite the observed variation in the quality and concentration of DNA crude samples obtained using different methods, their functionality in the downstream application was surprisingly good. It must be noted here that the concentration of DNA in the samples used for the testing of the DNA extraction method was equalized with distilled water according to the results of concentration tests prior to the molecular marker analyses (the final concentration was approximately 9 ng/μl) and that step was probably crucial for the obtained results. However, the resulting gel images (Figs. S3 in Online Resource 3 and S4 in Online Resource 4) showed no differences between the four methods, and the bands were clearly visible for all of them.

The objective for the development of the useful CAPS marker for the detection of mutant forms of winter rapeseed is to find a simple and quick method that would facilitate the breeding by analyzing a large number of samples. To achieve this aim, the simplified procedure was tested (see Materials and Methods). In the modified method, the purification step after the amplification of the *BnaA.FAD2* gene fragment and before its digestion with restriction enzyme was omitted. It was found that the results obtained using the simplified method are consistent with those obtained using the standard method (Fig. S5 in Online Resource 5).

**Fig. 3:**
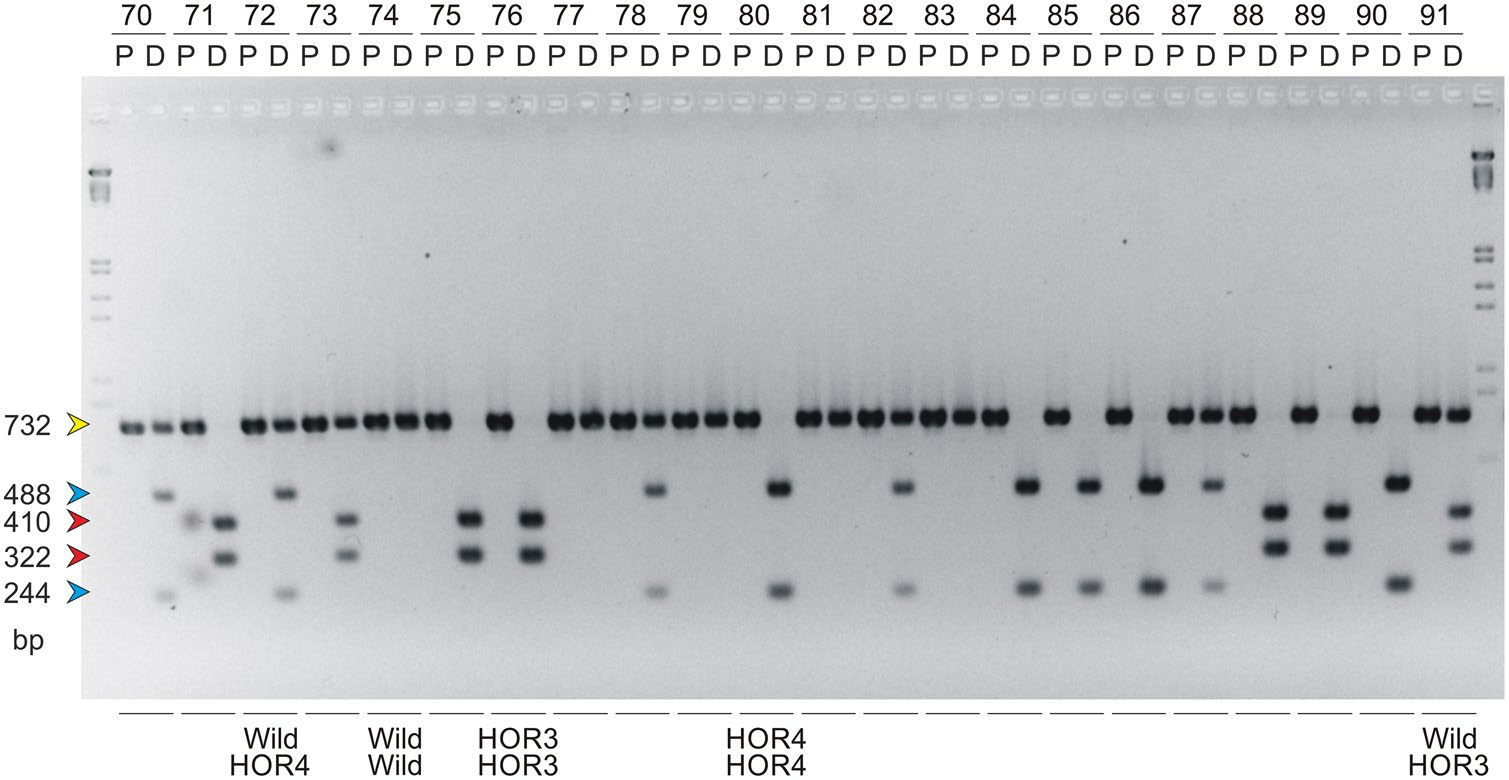
An example of results obtained using CAPS marker analyses for the rapeseed breeding lines in 2014. For each analyzed plant, two samples (designated with letters P and D, which are explained in Fig. 2) representing two steps of the CAPS protocol were applied on the gel. The numbers above each pair of lanes refer to the description of the plants delivered by breeders in 2014. The arrows indicate all five characteristic DNA fragments observed on the agarose gel, and their colors correspond to the colors used for the display of each mutation shown in Fig. 1. The five most frequent band patterns (in accordance with Table 1) are designated below each pair of lanes with the names of the specific alleles of the *BnaA.FAD2* gene that are present in the studied plant (these are “HOR3” and “HOR4” for the mutated alleles and “Wild” for the wild-type alleles, respectively) (color figure online)

The ability to distinguish between heterozygotes and homozygotes using the CAPS marker was also checked. The idea was to use mixed samples containing DNA from two different homozygous plants (mutated and wild-type forms) prepared to simulate the heterozygous one. For the HOR3 type mutation, the initial test was unsuccessful due to the problem related to the amplification of the mixed sample (Fig. S6 in Online Resource 6); however, in later experiments on the breeding material, the evidence that heterozygotes can be detected was obtained (Fig. 3 and Fig. S10 in Online Resource 10). Comparable analyses performed for the HOR4 type mutation yielded much better results (Fig. S7 in Online Resource 7). The CAPS marker analysis was successful both for mixed samples and for the real heterozygotes that were also found among the studied winter rapeseed lines. During the subsequent experiments on the breeding material, we found that many plants with the HOR4 type mutation exhibit heterozygotic state of the *BnaA.FAD2* gene (Fig. 3 and Figs. S9–S11 in Online Resources 9–11).

In addition to the molecular marker analyses, the seeds of some studied plants were analyzed using gas chromatography to determine the actual fatty acid content. Other important agronomic characteristics were also observed. With some exceptions, the plants carrying the mutated alleles of the *BnaA.FAD2* gene tend to have a higher amount of oleic acid in the seeds than the plants having wild-type ones, as shown in Table S1 in Online Resource 12.

Some of the analyzed samples were derived from the new series of mutagenesis experiments. These plants exhibited very high levels of oleic acid content in seeds (data not shown), which could be the result of some new mutations in the *BnaA.FAD2* gene or elsewhere in the genome. The present analyses showed that the tested CAPS marker cannot detect these new mutations (Fig. S8 in Online Resource 8). However, we found more plants exhibiting a high level of oleic acid content in seeds, which were not detected as mutants. These are, for example, the high oleic acid recombinant plants that were initially analyzed with the CAPS marker in the very early stage of our experiments (samples 12–14 of the basic set of 24 rapeseed lines, Fig. 2). They were derived from the ‘Contact’ cultivar, which is the alternative source of high oleic acid content in rapeseed. We suppose, that in this case, the high oleic acid content character of these recombinant plants also originated from the ‘Contact’ cultivar.

The new CAPS marker was extensively used for the MAS of winter rapeseed. The first *B. napus* plants were analyzed in 2011 (samples 6–11 and 16–24 of the basic set of 24 rapeseed lines, Fig. 2). For some samples, no products were obtained using the CAPS marker, probably due to the inhibition of PCR. Finally, by using both SCAR marker and the new CAPS marker, among the 15 studied plants, 6 homozygotic wild-type lines, 4 homozygotic HOR3 type lines, and 5 homozygotic HOR4 type lines were found.

In 2013, there were 50 *B. napus* genotypes analyzed using the CAPS marker. Among the studied plants, 11 homozygotic wild-type lines, 14 homozygotic HOR3 type lines, 17 homozygotic HOR4 type lines, and 8 heterozygotic lines with the mutated allele of HOR4 type were found (an example of these results is shown in Fig. S9 in Online Resource 9).

In 2014, there were 91 *B. napus* genotypes analyzed using the CAPS marker. Among the studied plants, we found 27 homozygotic wild-type lines, 5 homozygotic HOR3 type lines, 42 homozygotic HOR4 type lines, and 16 heterozygotic lines, of which 14 exhibited the mutated allele of HOR4 type and 2 possessed the mutated allele of HOR3 type (an example of these results is shown in Fig. 3). One sample showed an uncertain result in that analysis. This was the first time that the HOR3 heterozygotic form was found and detected experimentally using the CAPS marker. All the most frequent band patterns for the presented CAPS marker are shown on the resulting image of the gel (Fig. 3).

In 2015, there were 32 *B. napus* genotypes analyzed using the CAPS marker. Among the studied plants, 12 homozygotic wild-type lines, 5 homozygotic HOR3 type lines, 10 homozygotic HOR4 type lines, and 5 heterozygotic lines with the mutated allele of HOR4 type were found (Fig. S10 in Online Resource 10).

In recent years, the breeders have focused more on the offspring of HOR4-M10464 mutant. For example, in 2016, among the 51 samples with the mutated alleles, only 5 samples possessed the HOR3 type mutation (4 homozygotic HOR3 type lines and 1 heterozygotic line with the mutated allele of HOR3 type), and another 46 samples were of the HOR4 type (21 homozygotic HOR4 type lines and 25 heterozygotic lines with the mutated allele of HOR4 type). During the analyses performed in 2016, among the 99 samples analyzed, 48 were of wild-type, and the respective plants could be rejected from the breeding process (an example of these results is shown in Fig. S11 in Online Resource 11); thus, the marker has become a valuable selection tool for breeders.

## Discussion

The results of the present study provide some information regarding the performance of the CAPS marker and allow us to choose the best option for performing routine analyses. The basic question was whether the data obtained from the molecular analysis are reliable enough for implementation and whether the marker recognizes the right gene. It is well known that the rapeseed genome is very complex [37], which is a common characteristic of genomes of all *Brassica* species [38]. The triplicated nature of the diploid *Brassica* genomes can be observed [39–42]. It is thought that these genomes originate from the paleohexaploid, which was then the subject of segmental loss and limited segmental duplication [38]. Probably, many rounds of ancient polyploidizations and subsequent diploidizations have led to the present structure of *Brassica* genomes, thus causing the observed multiplication of many genes [38, 43–48]. Even 21 segments from the model *Arabidopsis thaliana* genome, representing almost its entirety, have been identified as being similar to the segments of the *B. napus* genome [37, 49]. The recent allopolyploidization leading to the origin of *B. napus* has increased the number of duplicated segments and has generated an additional duplication of many genes coming from the A and C genomes. The evidence that such multiplication arising from the segmental duplication exists for *B. napus* fatty acid desaturase genes has also been found [14]. This kind of homoeologous genes [50] may be very similar to each other, as they come from the very closely related diploid species. The rapeseed genome is evolving even more rapidly than the simple diploid *Brassica* genomes, as the recent allopolyploidization has led to the massive appearance of homoeologous chromosome segments. This in turn may cause many structural rearrangements due to the incorrect chromosome pairing during meiosis in the allopolyploid plant. The resulting offspring genomes exhibit the mosaic of the widespread structural variations (SV), and there is some evidence that the genome evolution caused by such variations is probably still ongoing in the present rapeseed varieties [37, 51]. All these evolutionary events that have increased the genome complexity make the molecular analyses of rapeseed genes much more difficult. Despite this complexity, there are some examples of successful design of molecular markers useful for breeding of this crop [15, 52–56], because it is still possible to identify some sequences that are specific for the particular gene. We thought that this is also the case for the present CAPS marker. The data obtained thus far seem to support that hypothesis, because the results of the molecular analyses were very clear and remained unaffected for subsequent generations (compare Fig. 3 and Figs. S9–S11 in Online Resources 9–11). Previous comparisons of the sequence that has led to the design of the marker with other *Brassica* sequences [25] have identified the amplified sequence of the *Bna.FAD2* gene as coming from the A genome, and not from the C genome; thus, our assumption that the analyzed gene is actually the *BnaA.FAD2* gene positioned on the A5 chromosome is based on these findings and on the fact that another locus from *B. rapa* is usually reported as non-functional in rapeseed [28, 29]. The clear experimental results presented here does not implicate any other hypothesis. However, the specificity of this marker for the A5 chromosome should finally be confirmed using the mapping population to clarify all doubts.

The results obtained using the CAPS marker was initially compared with those obtained previously using SCAR markers [30]. The analysis was performed using the same basic set of 24 *B. napus* lines and cultivars for better comparison (compare Fig. 2 and Fig. S2 in Online Resource 2). The results seem to be consistent with each other, and this was the first proof that the mutations are determined correctly using the developed CAPS marker. Both markers were designed on the basis of the same sequencing experiment [25], but the consistency of the results confirms the appropriate rationale underlying their design.

The procedure of the CAPS marker analysis was thoroughly tested. Four different methods of DNA extraction were examined for the use in this analysis. The results of the quality and concentration tests showed many differences between the samples obtained using various procedures, but the final results of the marker analyses seem to be immune to these differences. The samples obtained from the quick method (with SDS) showed the worse parameters when examining their test results. These samples exhibited lower DNA concentration, and the calculated A_260_/A_280_ and A_260_/A_230_ ratios were incorrect for many of them. It seems that this procedure could result in some protein contamination as well as poor efficiency of collecting pure genomic DNA. Nevertheless, the obtained gel images for these DNA samples showed some traces of intact DNA product (data not shown). These traces must have been enough for the subsequent CAPS analyses, because the results are consistent when compared with the standard CTAB method (Fig. S4 in Online Resource 4). The worse purity results of the samples obtained using the automatic FastPrep / FastDNA SPIN Kit procedure seem to be quite normal, taking into account that these samples were obtained using the column-based kit. In accordance with the Thermo Scientific technical bulletin [57], the lowest A_260_/A_230_ ratio may be the result of the residual guanidine thiocyanate originating from the Binding Matrix suspension of the FastDNA® SPIN Kit (MP Biomedicals). Nevertheless, these samples also gave the correct results of the CAPS marker compared with the automatic FastPrep / CTAB method, thus confirming that the guanidine thiocyanate impurities do not influence the downstream CAPS marker procedure (Fig. S3 in Online Resource 3). The obtained results showed that the method is only slightly affected by the quality of the extracted DNA. The basic step that may suffer from some impurities is the PCR itself. As the procedure assumes that at least two samples are finally presented on the 1.8% agarose gel (undigested and digested PCR product) it is easy to check whether the amplification was successful. Therefore, all the tested DNA extraction procedures can be potentially used for the studied CAPS marker. However, one must consider that by using the quick method (with SDS) or column-based methods, the risk that the 732 bp product will not be amplified correctly is slightly higher. Even if we can conclude that the cheapest, least laborious, and quickest method is preferred, it is still better to use more reliable and less risky method to avoid the wastage of time and reagents in case the analysis fails. Hence, we recommend to use the standard CTAB method (that may eventually be replaced by the automatic FastPrep / CTAB procedure) if the time is not the main limiting factor. All the other results presented here relied on the standard DNA extraction procedure.

Although the original CAPS marker procedure proposed by Konieczny and Ausubel [31] assumes only two basic steps (amplification and digestion with restriction enzyme), our initial standard protocol had an additional purification step included. The reason for using this step, which was quite an expensive one, was to assure that the digestion reaction is not affected by some reagents from the amplification step. It is known that some organic compounds may cause star activity, loss of specificity, or even inhibit the activity of selected restriction endonucleases [58–60]. Testing the marker was a good opportunity to verify the behavior of the *Fsp*BI restriction enzyme (Fermentas/Thermo Scientific) in this specific context. After the comparison of the results obtained using the initial method with the purification step and the simplified method without that step (similar to the method proposed by Konieczny and Ausubel [31]), we found that the outcomes obtained with both methods are identical (Fig. S5 in Online Resource 5). Based on these findings, we decided not to use the additional purification step in our recommended protocol. As the DNA purification kits are expensive, omitting this step will make the procedure cheaper and less time consuming, and at the same time, the fact that no interference with the digestion step exists was also proved.

Potentially, the main advantage of the present marker over the previous ones was the ability to distinguish between homozygotes and heterozygotes [31]. However, we thought that it was necessary to verify this ability through experiments. Because we could not foresee whether any genotypes exhibit the heterozygotic state of the *BnaA.FAD2* gene among the studied plant materials, we decided to fasten the process and prepared special mixed samples to simulate its heterozygosity. On the basis of previous analyses, the DNA samples from homozygous forms of all three types (HOR3, HOR4 and wild) were precisely identified. These samples were then used to prepare mixed DNA samples that were analyzed using the CAPS marker. To avoid preferential amplification of one allele, which was previously discussed by Konieczny and Ausubel [31] for the CAPS markers, special care was taken to apply an equal amount of each homozygous DNA sample. The initial results of this test (Figs. S6 in Online Resource 6 and S7 in Online Resource 7) were only partially successful, as the mixed sample with HOR3 type mutated allele failed to amplify correctly, resulting in faint bands. Nevertheless, the HOR4 type simulated heterozygote was detected in accordance with our expectations. Regarding the failed analysis of the HOR3 type heterozygote, we had to wait until the relevant plant appeared among the breeding plant material, and finally, positive results for that case were also confirmed experimentally (Fig. 3).

The phenotypic data were very useful for the verification of the results obtained using the CAPS marker. The results of both analyses seem to be generally consistent with each other, showing some relatedness (Table S1 in Online Resource 12) with the higher oleic acid content in seeds of plants carrying the mutated alleles of the *BnaA.FAD2* gene, although some exceptions from this general rule were also observed (see the discussion below on the analyses of other rapeseed sources exhibiting increased oleic acid content in seeds and on the breeding of the PN-880 recombined line using MAS). At present, no statistical analysis of the phenotypic data has been performed; however, we plan to make such a detailed comparison in the future. Hopefully, it will provide the statistical evidence for the observed interdependence and show the impact of single mutated allele of heterozygotes versus doubled alleles of homozygotes.

The comparison of sequencing data from the studied mutants and from other high oleic acid mutants [15, 28] has proved that various mutations of the *BnaA.FAD2* gene may lead to the similar phenotypic effect. It was experimentally demonstrated that the marker is specific only to the selected mutations described earlier [8, 9, 25–27]. Two other sources of rapeseed with increased oleic acid content in the seed were also analyzed with the CAPS marker showing the wild-type genotype (samples 12–14 in Fig. 2 and the results shown in Fig. S8 in Online Resource 8). Some of these plants exhibited even higher oleic acid content than the original mutant plants presented in Table S1 in Online Resources 12 (data not shown). It can be assumed that these genotypes have some altered forms of the Δ12 oleate desaturase genes coming both from A genome and from the homoeologous segment in C genome. The increased amount of oleic acid in the seeds of these plants may be due to the blocked expression of both copies of rapeseed desaturase genes or the disturbed activity of the resulting enzymes derived from both subgenomes. The relevant alleles seem to be structurally different from those of the mutants described in this study. The present results and our initial analyses [61, 62] proved that the altered *BnaA.FAD2* gene alleles, which originate from the alternative sources cannot be identified with the described CAPS marker even if they show the same phenotypic effect (the observed band profile is like that for the wild-type form) (samples 12–14 in Fig. 2 and the results shown in Fig. S8 in Online Resource 8). The other possible explanation for the high oleic acid content in these plants is the presence of some other genes associated with the regulation of the oleic acid level in the seeds. The analysis of such genes is out of the scope of the studied specific CAPS marker.

**Fig. 4:**
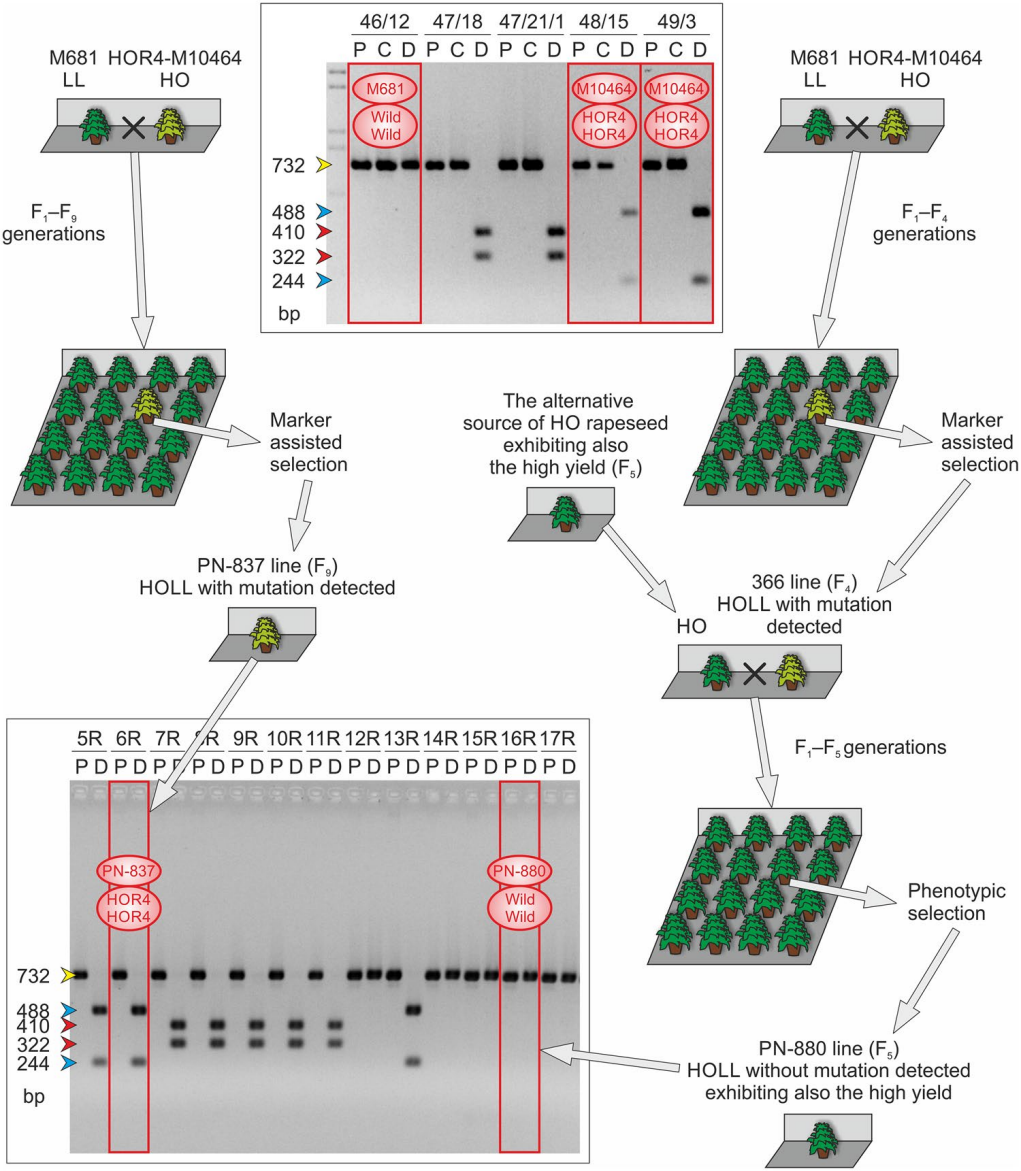
The breeding scheme of two HOLL breeding lines (PN-880 and PN-837) exhibiting different CAPS marker genotypes, showing the possible explanation for the observed differences. The relevant CAPS analyses of the progenitor lines and the resulting HOLL lines are also shown (the results for the discussed progenitors and HOLL lines are indicated using red rectangles and the detected alleles of the *BnaA.FAD2* gene are named “HOR4” for the mutated alleles and “Wild” for the wild-type alleles). For each analyzed plant, two or three samples (designated with letters P, C, and D, which are explained in Fig. 2) representing two or three steps of the CAPS protocol were applied on the gel. The numbers above each group of lanes refer to the description of the plants delivered by breeders. The arrows on the left side of each gel image indicate the DNA fragments observed on the agarose gel, and their colors correspond to the colors used for the display of each mutation shown in Fig. 1 (color figure online)

In parallel with the testing of all parameters and assessing the various modifications in the CAPS marker protocol that were mentioned above, the CAPS marker was also used as a tool for breeding of the new winter rapeseed varieties (samples 6–11 and 16–24 of the basic set of 24 rapeseed lines in Fig. 2 and results shown in Figs. 3–4 and in Figs. S9–S11 in Online Resources 9–11). Because the CAPS marker has been in the development stage during these years, the majority of the breeding material was analyzed using both biochemical and molecular methods. It was therefore a good opportunity for us to test the performance of the marker on the generation-to-generation basis by comparing the results of both methods. Additionally, the comparison of the obtained marker data with the respective breeding schemes obtained from the breeders confirmed the presence of the mutant alleles in successive generations, which was also very valuable information for us. Between 2011 and 2018, the mutated alleles of HOR4 or HOR3 type and the wild-type alleles were observed in a large number of studied materials. In successive generations, all five basic band profiles were detected using the new CAPS marker. Furthermore, most of the lines that were bred for the high level of oleic acid in seeds became positive for the HOR4 type allele. This mutation type has been finally chosen by breeders, despite the better yield observed for the second one (compare both mutants in Table S1 in Online Resource 12). The reason to choose the HOR4 type mutant was mainly the better stability of the oleic acid level than that of another mutant. Hence, more HOR4 type mutant alleles were detected recently. The revealed mutant alleles occurred in both homozygotic and heterozygotic states, depending on the test line. Many of the tested samples also exhibited the wild-type alleles, and in such cases, the respective plants could be rejected from the breeding process, making it much less laborious.

Even though the present marker fulfilled our requirements as the precise molecular marker in the majority of cases, showing some relatedness between the high oleic acid content in seeds and the presence of the mutated alleles of the *BnaA.FAD2* gene, some cases were noted where the obtained data remained in disagreement. For example, despite maintaining the very high content of oleic acid in seeds (as determined by the gas chromatography analysis), the presence of mutated alleles of the *BnaA.FAD2* gene has not been detected in the PN-880 line using the allele-specific CAPS marker (Fig. 4, Table S1 in Online Resource 12). The characteristic of this HOLL line is that at a certain stage, its progenitor was crossed with rapeseed materials from another breeding program, which may carry the alternative source of high oleic acid content in seeds similar to that described above (samples 12–14 in Fig. 2 and the results shown in Fig. S8 in Online Resource 8). This would explain the reason for very high oleic acid content in the successive generations. Probably at the time of selection (based mainly on the biochemical analyses), the plants with the highest oleic acid content, which originated from the alternative source, were preferably selected, and the forms containing mutations identified by the CAPS marker were rejected.

On the other hand, a good example of the successful selection of the winter rapeseed lines having high content of oleic acid in seeds is the HOLL type PN-837 line, which was obtained after crossing of the high oleic acid HOR4 type mutant line and the low linolenic acid mutant line (M681) (Fig. 4, Table S1 in Online Resource 12). In this case, a different situation was observed than in the case of the PN-880 line mentioned above. The only source of high oleic acid content in this material was the HOR4-M10464 mutant. By using the molecular marker, the presence of the mutant alleles in successive generations was confirmed. In F_9_ generation, stable lines of HOLL type were obtained, with a better agronomic value. The presence of two mutated alleles of the *BnaA.FAD2* gene (the HOR4 type mutated homozygote for this gene) resulted in the high content of oleic acid, i.e., 76%. The use of the CAPS marker made the selection possible in terms of other important features while maintaining alleles associated with the high oleic acid content in seeds.

On the basis of the present results, we can say that the well-established standard CTAB method of DNA extraction and the simplified, two-step (amplification/digestion) procedure for the CAPS marker specific for two mutations in the *BnaA.FAD2* gene are the best options to obtain reliable and clear results of the analysis. We recommend to have the undigested (control) and digested samples from the same plant placed side by side on the agarose gel for better control of the reactions. The allele-specific CAPS marker described here allows for an effective selection of breeding lines of winter rapeseed with the increased content of oleic acid in seeds originating from one of two initial mutated forms of winter rapeseed (HOR3-M10453 or HOR4-M10464). The marker was successfully used for such selection and can potentially assure the breeders of the purity of their HOLL lines on every step of the breeding process as well as after the registration of the resulting HOLL varieties. However, the fact that the marker may be used only for the detection of two specific types of mutations in the *BnaA.FAD2* gene, which were described above, should also be considered. It is impossible to detect other alleles of this gene, even if they show the same phenotypic effect. The marker is also useless for the analysis of other genes associated with the content of oleic acid in the seeds, including the homoeologous Δ12 oleate desaturase gene located in the C genome of rapeseed. Nevertheless, the use of this molecular marker in future breeding programs of winter rapeseed will hopefully fasten the progress in the breeding of new varieties with high oleic acid content in seeds.

## Electronic supplementary material

Below is the link to the electronic supplementary material.

Supplementary file1 (PDF 260 kb)

Supplementary file2 (PDF 1515 kb)

Supplementary file3 (PDF 1576 kb)

Supplementary file4 (PDF 1535 kb)

Supplementary file5 (PDF 1924 kb)

Supplementary file6 (PDF 1475 kb)

Supplementary file7 (PDF 1450 kb)

Supplementary file8 (PDF 1471 kb)

Supplementary file9 (PDF 1630 kb)

Supplementary file10 (PDF 1940 kb)

Supplementary file11 (PDF 1799 kb)

Supplementary file12 (PDF 449 kb)

## Data Availability

All data generated or analyzed during this study are included in this published article and its supplementary information files.
