# Supplementary material for: Cleaved amplified polymorphic sequences (CAPS) marker for identification of two mutant alleles of the rapeseed BnaA.FAD2 gene
Source: Mol Biol Rep. 2020 Sep 26;47(10):7607–21. doi: 10.1007/s11033-020-05828-2 (PMC7588397; doi:10.1007/s11033-020-05828-2)
Supplement: Supplementary file 1 — Supplementary file1 (PDF 260 kb) [file 11033_2020_5828_MOESM1_ESM.pdf]

```
BnaA.FAD2.a (Yang)     ATGGGTGCAGGTGGAAGAATGCAAGTGTCTCCTCCCTCCAAAAAGTCTGAAACCGACAACATCAAGCGCGTACCCTGCGAGACACCGCCCTTCACTGTCG 100
BraA.FAD2.a (Yang)     ATGGGTGCAGGTGGAAGAATGCAAGTGTCTCCTCCCTCCAAAAAGTCTGAAACCGACAACATCAAGCGCGTACCCTGCGAGACACCGCCCTTCACTGTCG 100
BnaA.FAD2.a (Zhang)    ATGGGTGCAGGTGGAAGAATGCAAGTGTCTCCTCCCTCCAAAAAGTCTGAAACCGACAACATCAAGTGCGTACCCTGCGAGACACCGCCCTTCACTGTCG 100
H3H4CON1 primer                               5'-AGTGTCTCCTCCCTCCAAAAA-3'

BnaA.FAD2.a (Yang)     GAGAACTCAAGAAAGCAATCCCACCGCACTGTTTCAAACGCTCGATCCCTCGCTCTTTCTCCTACCTCATCTGGGACATCATCATAGC--CTCCTGCTTC 198
BraA.FAD2.a (Yang)     GAGAACTCAAGAAAGCAATCCCACCGCACTGTTTCAAACGCTCGATCCCTCGCTCTTTCTCCTACCTCATCTGGGACATCATCATAGC--CTCCTGCTTC 198
BnaA.FAD2.a (Zhang)    GAGAACTCAAGAAAGCAATCCCACCGCACTGTTTCAAACGCTCGATCCCTCGCTCTTTCTCCTACCTCATCTGGGACATCATCATAGC--CTCCTGCTTC 198

BnaA.FAD2.a (Yang)     TACTACGTCGCCACCACTTACTTCCCTCTCCTCCCTCACCCTCTCTCCTACTTCGCCTGGCCTCTCTACTGGGCCTGCCAGGGCTGCGTCCTAACCGGCG 298
BraA.FAD2.a (Yang)     TACTACGTCGCCACCACTTACTTCCCTCTCCTCCCTCACCCTCTCTCCTACTTCGCCTGGCCTCTCTACTGGGCCTGCCAGGGCTGCGTCCTAACCGGCG 298
BnaA.FAD2.a (Zhang)    TACTACGTCGCCACCACTTACTTCCCTCTCCTCCCTCACCCTCTCTCCTACTTCGCCTGGCCTCTCTACTGGGCCTGCCAGGGCTGCGTCCTAACCGGCG 298
HOR4 type mutation [8] (G→A at position 269)                                                           CTAG

BnaA.FAD2.a (Yang)     TCTGGGTCATAGCCCACGAGTGCGGCCACCACGCCTTCAGCGACTACCAGTGGCTGGACGACACCGTCGGCCTCATCTTCCACTCCTTCCTCCTCGTCCC 398
BraA.FAD2.a (Yang)     TCTGGGTCATAGCCCACGAGTGCGGCCACCACGCCTTCAGCGACTACCAGTGGCTGGACGACACCGTCGGCCTCATCTTCCACTCCTTCCTCCTCGTCCC 398
BnaA.FAD2.a (Zhang)    TCTGGGTCATAGCCCACGAGTGCGGCCACCACGCCTTCAGCGACTACCAGTGGCTGGACGACACCGTCGGCCTCATCTTCCACTCCTTCCTCCTCGTCCC 398
HOR3 type mutation [8] (C→T at position 346)                                   CTAG

BnaA.FAD2.a (Yang)     TTACTTCTCCTGGAAGTACAGTCATCGACGCCACCATTCCAACACTGGCTCCCTCGAGAGAGACGAAGTGTTTGTCCCCAAGAAGAAGTCAGACATCAAG 498
BraA.FAD2.a (Yang)     TTACTTCTCCTGGAAGTACAGTCATCGACGCCACCATTCCAACACTGGCTCCCTCGAGAGAGACGAAGTGTTTGTCCCCAAGAAGAAGTCAGACATCAAG 498
BnaA.FAD2.a (Zhang)    TTACTTCTCCTGGAAGTACAGTCATCGACGCCACCATTCCAACACTGGCTCCCTCGAGAGAGACGAAGTGTTTGTCCCCAAGAAGAAGTCAGACATCAAG 498

BnaA.FAD2.a (Yang)     TGGTACGGCAAGTACCTCAACAACCCTTTGGGACGCACCGTGATGTTAACGGTTCAGTTCACTCTCGGC----TGGCCTTTGTACTTAGCCTTCAACGTC 594
BraA.FAD2.a (Yang)     TGGTACGGCAAGTACCTCAACAACCCTTTGGGACGCACCGTGATGTTAACGGTTCAGTTCACTCTCGGC----TGGCCTTTGTACTTAGCCTTCAACGTC 594
BnaA.FAD2.a (Zhang)    TGGTACGGCAAGTACCTCAACAACCCTTTGGGACGCACCGTGATGTTAACGGTTCAGTTCACTCTCGGC----TGGCCTTTGTACTTAGCCTTCAACGTC 594
DMS100 type mutation [15] (C→T at position 553)                                    CGGTTTAGTTC
SW Hickory type mutation [28] (AGCC insertion at position 567-568)                         TCGGCAGCCTGGCC
```

BnaA.FAD2.a (Yang) TCGGGGAGACCTTACGACGGCGGCTTCGCTTGCCATTTCCACCC**C**AACGCTCCCATCTACAACGACCGTGAGCGTCTCCAGATATACATCTCCGACGCTG 694
BraA.FAD2.a (Yang) TCGGGAAGACCTTACGACGGCGGCTTCGCTTGCCATTTCCACCC**T**AACGCTCCCATCTACAACGACCGTGAGCGTCTCCAGATATACATCTCCGACGCTG 694
BnaA.FAD2.a (Zhang) TCGGGGAGACCTTACGACGGCGGGTTCGCTTGCCATTTCCACCC**C**AACGCTCCCATCTACAACGACCGTGAGCGTCTCCAGATATACATCTCCGACGCTG 694

BnaA.FAD2.a (Yang) GCATCCTCGCCGTCTGCTACGGTCTCTACCGCTACGCTGCTGTCCAAGGAGTTGCCTCGATGGTCTGCTTCTACGGAGT**T**CC**T**CTTCTGAT**T**GTCAACGG 794
BraA.FAD2.a (Yang) GCATCCTCGCCGTCTGCTACGGTCTCTACCGCTACGCTGCTGTCCAAGGAGTTGCCTCGATGGTCTGCTTCTACGGAGT**C**CC**G**CTTCTGAT**A**GTCAACGG 794
BnaA.FAD2.a (Zhang) GCATCCTCGCCGTCTGCTACGGTCTCTACCGCTACGCTGCTGTCCAAGGAGTTGCCTCGATGGTCTGCTTCTACGGAGT**T**CC**T**CTTCTGAT**T**GTCAACGG 794
H3H4CON2 primer 3′-**AGGTTCCTCAACGGAGCTA**-5′

BnaA.FAD2.a (Yang) GTTCTTAGTTTTGATCACTTACTTGCAGCACACGCATCCTTCCCTGCCTCACTA**T**GA**C**TCGTCTGAGTGGGATTGGTTGAGGGGAGC**T**TTGGC**C**ACCGTT 894
BraA.FAD2.a (Yang) GTTCTTAGTTTTGATCACTTACTTGCAGCACACGCATCCTTCCCTGCCTCACTA**C**GA**T**TCGTCTGAGTGGGATTGGTTGAGGGGAGC**G**TTGGC**T**ACCGTT 894
BnaA.FAD2.a (Zhang) GTTCTTAGTTTTGATCACTTACTTGCAGCACACGCATCCTTCCCTGCCTCACTA**T**GA**C**TCGTCTGAGTGGGATTGGTTGAGGGGAGC**T**TTGGC**C**ACCGTT 894

BnaA.FAD2.a (Yang) GACAGAGACTACGG**A**ATCTTGAACAAGGTCTTCCACAATATCACGGACACGCACGTGGCGCATCACCTGTTCTCGACCA**T**GCCGCATTATCA**T**GCGATGG 994
BraA.FAD2.a (Yang) GACAGAGACTACGG**G**ATCTTGAACAAGGTCTTCCACAATATCACGGACACGCACGTGGCGCATCACCTGTTCTCGACCA**C**GCCGCATTATCA**C**GCGATGG 994
BnaA.FAD2.a (Zhang) GACAGAGACTACGG**A**ATCTTGAACAAGGTCTTCCACAATATCACGGACACGCACGTGGCGCATCACCTGTTCTCGACCA**T**GCCGCATTATCA**T**GCGATGG 994

BnaA.FAD2.a (Yang) AAGCTAC**G**AAGG**C**GATAAAGCCGATACTG-GGAGAGTATTATCAGTTCGATGGGACGCCGGTGGTTAAGGCGATGTGGAGGGAGGCGAAGGAGTGTATCT 1093
BraA.FAD2.a (Yang) AAGCTAC**C**AAGG**C**GATAAAGCCGATACTG-GGAGAGTATTATCAGTTCGATGGGACGCCGGTGGTTAAGGCGATGTGGAGGGAGGCGAAGGAGTGTATCT 1093
BnaA.FAD2.a (Zhang) AAGCTAC**G**AAGG**T**GATAAAGCCGATACTG-GGAGAGTATTATCAGTTCGATGGGACGCCGGTGGTTAAGGCGATGTGGAGGGAGGCGAAGGAGTGTATCT 1093

BnaA.FAD2.a (Yang) ATGTGGAACCGGACAGGCAAGGTGAGAAGAAAGGTGTGTTCTGGTACAACAATAAGTTATGA 1155
BraA.FAD2.a (Yang) ATGTGGAACCGGACAGGCAAGGTGAGAAGAAAGGTGTGTTCTGGTACAACAATAAGTTATGA 1155
BnaA.FAD2.a (Zhang) ATGTGGAACCGGACAGGCAAGGTGAGAAGAAAGGTGTGTTCTGGTACAACAATAAGTTATGA 1155

Fig. S1 The alignment of the sequences of the *BnaA.FAD2.a* (Yang) gene from *B.napus*, the *BraA.FAD2.a* (Yang) gene from *B.rapa* [28] and the *BnaA.FAD2.a* (Zhang) gene from *B.napus* [18] (GenBank accession AY577313). The differences are indicated with green color and the underlined nucleotides represent the codons in the coding sequence. The sequence of the *BnaA.FAD2.a* (Zhang) gene was used for amplification of the Δ12 oleate desaturase gene from genome A (located on the chromosome A5), and therefore, this sequence is the basis for design of described CAPS markers. The positions of HOR3 and HOR4 type mutations in the studied gene, and the allelic forms that are specific to the HO mutants [8], are indicated using the color scheme that corresponds to the colors used for the display of each mutation shown in Fig. 1. The sequences of the H3H4CON1 (forward) and the H3H4CON2 (reverse) primers used for the amplification of the specific fragment of the *BnaA.FAD2* gene are also shown and the corresponding sequences of the gene are indicated with yellow color. In addition, the positions of other mutations in this gene, discovered and described by other authors [15, 28] are also indicated for comparison

Molecular Biology Reports

Cleaved amplified polymorphic sequences (CAPS) marker for identification of two mutant alleles of the rapeseed *BnaA.FAD2* gene

Marcin Matuszczak, Stanisław Spasibionek, Katarzyna Gacek, Iwona Bartkowiak-Broda

Corresponding author: Marcin Matuszczak

Plant Breeding and Acclimatization Institute, National Research Institute, Research Division in Poznań, Poland

E-mail: marmat@nico.ihar.poznan.pl
