# Supplementary material for: Cleaved amplified polymorphic sequences (CAPS) marker for identification of two mutant alleles of the rapeseed BnaA.FAD2 gene
Source: Mol Biol Rep. 2020 Sep 26;47(10):7607–21. doi: 10.1007/s11033-020-05828-2 (PMC7588397; doi:10.1007/s11033-020-05828-2)
Supplement: Supplementary file 2 — Supplementary file2 (PDF 1515 kb) [file 11033_2020_5828_MOESM2_ESM.pdf]

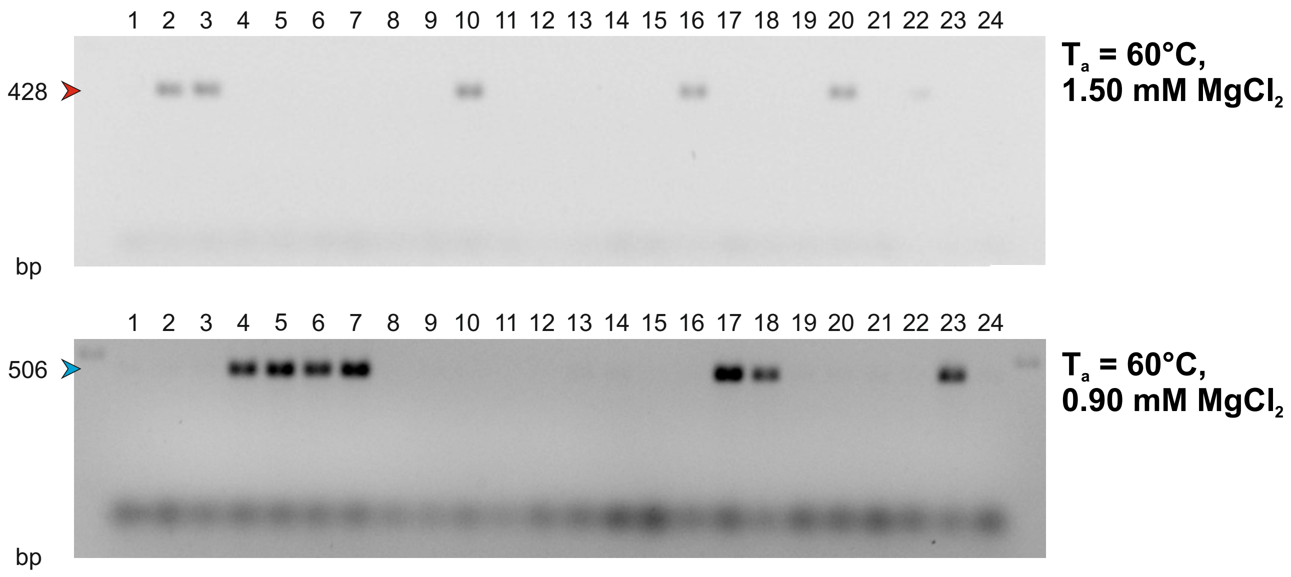

Fig. S2 The results of the analyses performed using the SCAR markers specific for the mutations in the *BnaA.FAD2* gene of rapeseed for the basic set of 24 rapeseed lines (see Materials and Methods for the description of each line). The arrows indicate the amplified DNA fragments, and their colors correspond to the colors used for the display of each mutation shown in Fig. 1 [30]

Molecular Biology Reports

Cleaved amplified polymorphic sequences (CAPS) marker for identification of two mutant alleles of the rapeseed *BnaA.FAD2* gene

Marcin Matuszczak, Stanisław Spasibionek, Katarzyna Gacek, Iwona Bartkowiak-Broda

Corresponding author: Marcin Matuszczak
Plant Breeding and Acclimatization Institute, National Research Institute, Research Division in Poznań, Poland
E-mail: marmat@nico.ihar.poznan.pl
