# Supplementary material for: Cleaved amplified polymorphic sequences (CAPS) marker for identification of two mutant alleles of the rapeseed BnaA.FAD2 gene
Source: Mol Biol Rep. 2020 Sep 26;47(10):7607–21. doi: 10.1007/s11033-020-05828-2 (PMC7588397; doi:10.1007/s11033-020-05828-2)
Supplement: Supplementary file 4 — Supplementary file4 (PDF 1535 kb) [file 11033_2020_5828_MOESM4_ESM.pdf]

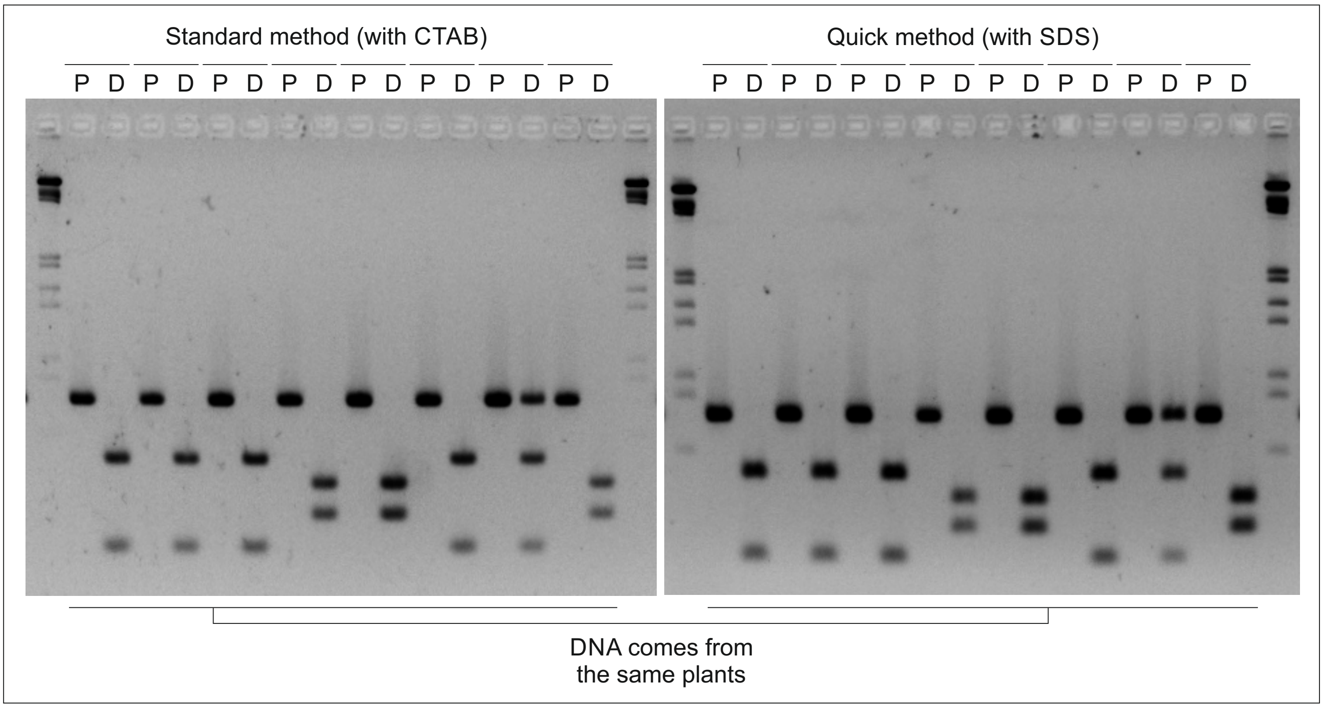

Fig. S4 Comparison of the CAPS marker results obtained for the same set of rapeseed plants using standard (with CTAB) and quick (with SDS) DNA extraction procedures. For each analyzed plant, two samples (designated with letters P and D, which are explained in Fig. 2) representing two steps of the CAPS protocol were applied on the gel

Molecular Biology Reports

Cleaved amplified polymorphic sequences (CAPS) marker for identification of two mutant alleles of the rapeseed *BnaA.FAD2* gene

Marcin Matuszczak, Stanisław Spasibionek, Katarzyna Gacek, Iwona Bartkowiak-Broda

Corresponding author: Marcin Matuszczak
Plant Breeding and Acclimatization Institute, National Research Institute, Research Division in Poznań, Poland
E-mail: marmat@nico.ihar.poznan.pl
