# Supplementary material for: Cleaved amplified polymorphic sequences (CAPS) marker for identification of two mutant alleles of the rapeseed BnaA.FAD2 gene
Source: Mol Biol Rep. 2020 Sep 26;47(10):7607–21. doi: 10.1007/s11033-020-05828-2 (PMC7588397; doi:10.1007/s11033-020-05828-2)
Supplement: Supplementary file 6 — Supplementary file6 (PDF 1475 kb) [file 11033_2020_5828_MOESM6_ESM.pdf]

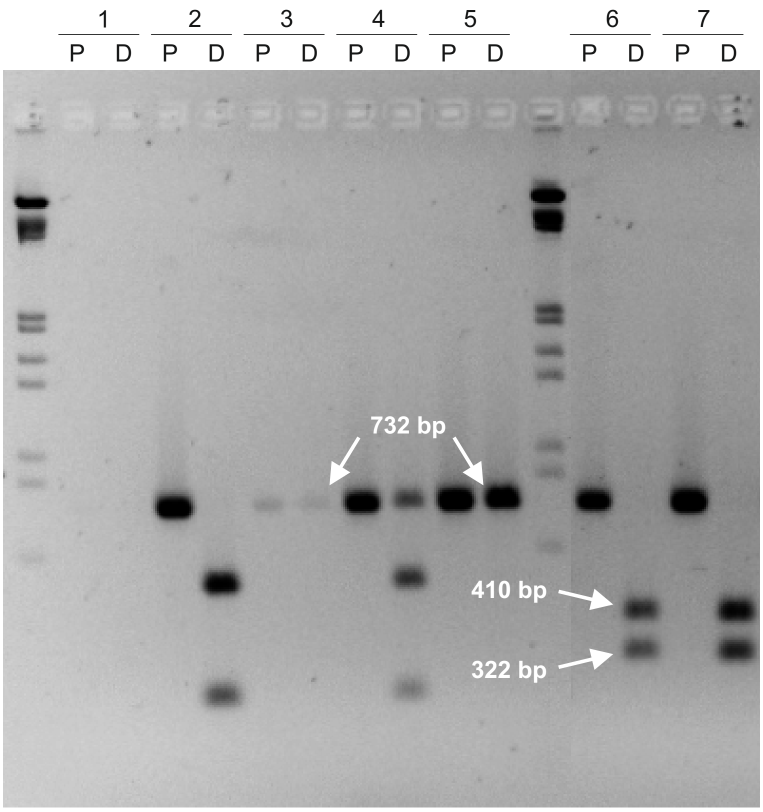

1—the sample coming from the HOR3 type homozygote (DNA was degraded or some inhibitors were present)
3—the mixture of the samples 1 and 5 (the simulation of the heterozygote with HOR3 type mutated allele)
2, 4—the samples coming from the HOR4 type homozygotic and heterozygotic plants (for comparison)
5—the sample coming from the wild-type homozygote (the ‚Monolit' cultivar)
6, 7—the samples coming from the other HOR3 type homozygotic plants (with correct results, for comparison)

Fig. S6 Results of testing the ability to detect heterozygous plants possessing both the HOR3 type mutated and wild-type alleles of the *BnaA.FAD2* gene of rapeseed by using the CAPS marker. All the tested DNA samples are described in the figure. For each analyzed DNA, two samples (designated with letters P and D, which are explained in Fig. 2) representing two steps of the CAPS protocol were applied on the gel. The white arrows indicate the characteristic bands of the HOR3 type mutated and wild-type alleles

Molecular Biology Reports

Cleaved amplified polymorphic sequences (CAPS) marker for identification of two mutant alleles of the rapeseed *BnaA.FAD2* gene

Marcin Matuszczak, Stanisław Spasibionek, Katarzyna Gacek, Iwona Bartkowiak-Broda

Corresponding author: Marcin Matuszczak
Plant Breeding and Acclimatization Institute, National Research Institute, Research Division in Poznań, Poland
E-mail: marmat@nico.ihar.poznan.pl
