# Supplementary material for: Cleaved amplified polymorphic sequences (CAPS) marker for identification of two mutant alleles of the rapeseed BnaA.FAD2 gene
Source: Mol Biol Rep. 2020 Sep 26;47(10):7607–21. doi: 10.1007/s11033-020-05828-2 (PMC7588397; doi:10.1007/s11033-020-05828-2)
Supplement: Supplementary file 7 — Supplementary file7 (PDF 1450 kb) [file 11033_2020_5828_MOESM7_ESM.pdf]

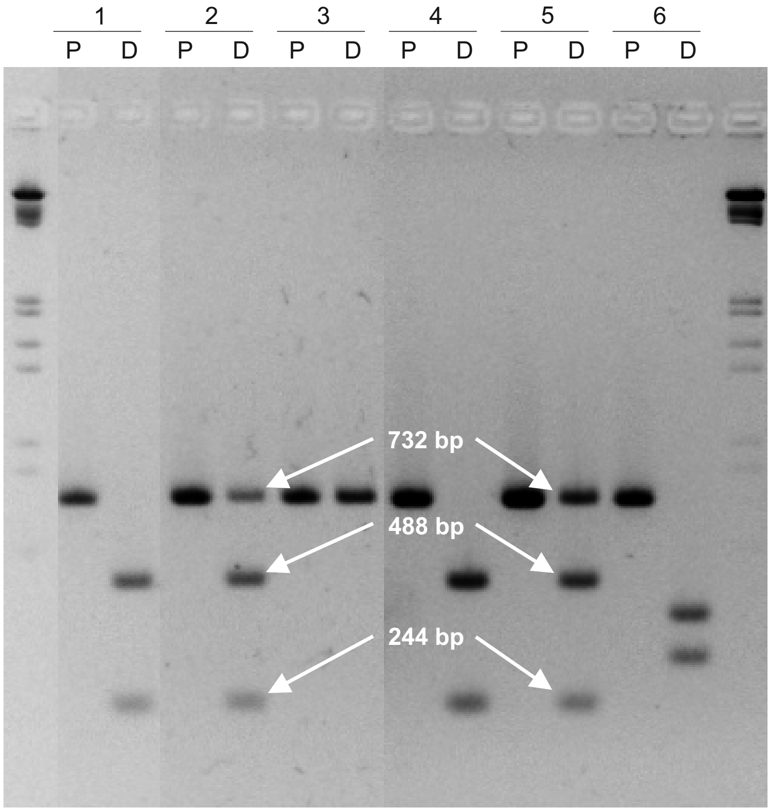

Fig. S7 Results of testing the ability to detect heterozygous plants possessing both the HOR4 type mutated and wild-type alleles of the *BnaA.FAD2* gene of rapeseed using the CAPS marker. All the tested DNA samples are described in the figure. For each analyzed DNA, two samples (designated with letters P and D, which are explained in Fig. 2) representing two steps of the CAPS protocol were applied on the gel. The white arrows indicate the characteristic bands of the HOR4 type mutated and wild-type alleles

Molecular Biology Reports

Cleaved amplified polymorphic sequences (CAPS) marker for identification of two mutant alleles of the rapeseed *BnaA.FAD2* gene

Marcin Matuszczak, Stanisław Spasibionek, Katarzyna Gacek, Iwona Bartkowiak-Broda

Corresponding author: Marcin Matuszczak
Plant Breeding and Acclimatization Institute, National Research Institute, Research Division in Poznań, Poland
E-mail: marmat@nico.ihar.poznan.pl
