# Supplementary material for: Cleaved amplified polymorphic sequences (CAPS) marker for identification of two mutant alleles of the rapeseed BnaA.FAD2 gene
Source: Mol Biol Rep. 2020 Sep 26;47(10):7607–21. doi: 10.1007/s11033-020-05828-2 (PMC7588397; doi:10.1007/s11033-020-05828-2)
Supplement: Supplementary file 8 — Supplementary file8 (PDF 1471 kb) [file 11033_2020_5828_MOESM8_ESM.pdf]

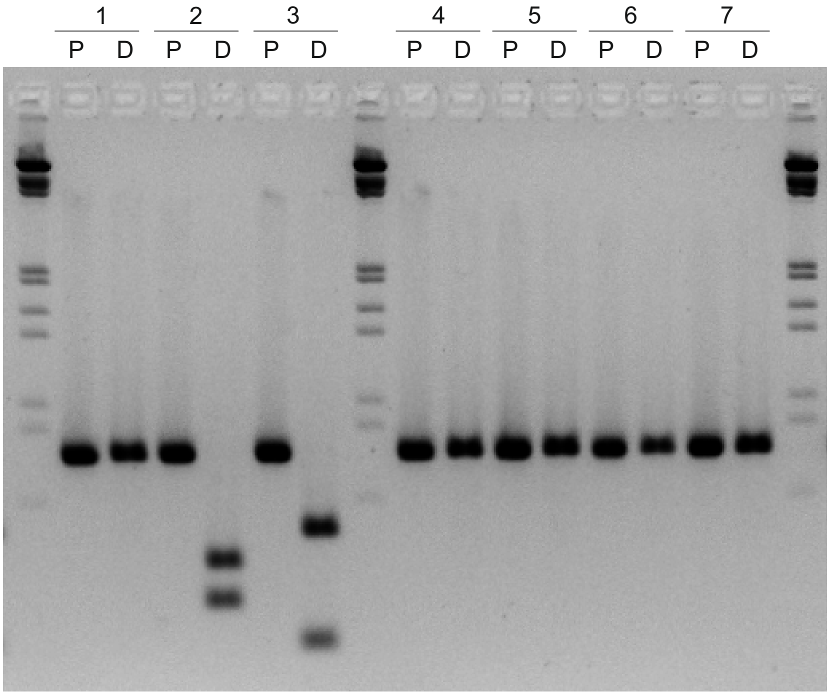

1–3—the control samples (wild, HOR3 and HOR4 type homozygote, respectively)
4–7—the samples coming from the plants that originated from the new series of mutagenesis experiments (124/2i/10—low-linolenic (LL), 150/5i/10—high-oleic (HO), 190/6i/10—HO, and 231/4i/10—HO, respectively)

Fig. S8 Results of testing the ability of the studied CAPS marker to detect the alternative source of high oleic acid content in rapeseed. The analyzed plants originated from the new series of mutagenesis experiments which could generate the new alleles of the *BnaA.FAD2* gene. All the tested DNA samples are described in the figure. For each analyzed plant, two samples (designated with letters P and D, which are explained in Fig. 2) representing two steps of the CAPS protocol were applied on the gel

Molecular Biology Reports

Cleaved amplified polymorphic sequences (CAPS) marker for identification of two mutant alleles of the rapeseed *BnaA.FAD2* gene

Marcin Matuszczak, Stanisław Spasibionek, Katarzyna Gacek, Iwona Bartkowiak-Broda

Corresponding author: Marcin Matuszczak
Plant Breeding and Acclimatization Institute, National Research Institute, Research Division in Poznań, Poland
E-mail: marmat@nico.ihar.poznan.pl
