# Supplementary material for: Cleaved amplified polymorphic sequences (CAPS) marker for identification of two mutant alleles of the rapeseed BnaA.FAD2 gene
Source: Mol Biol Rep. 2020 Sep 26;47(10):7607–21. doi: 10.1007/s11033-020-05828-2 (PMC7588397; doi:10.1007/s11033-020-05828-2)
Supplement: Supplementary file 10 — Supplementary file10 (PDF 1940 kb) [file 11033_2020_5828_MOESM10_ESM.pdf]

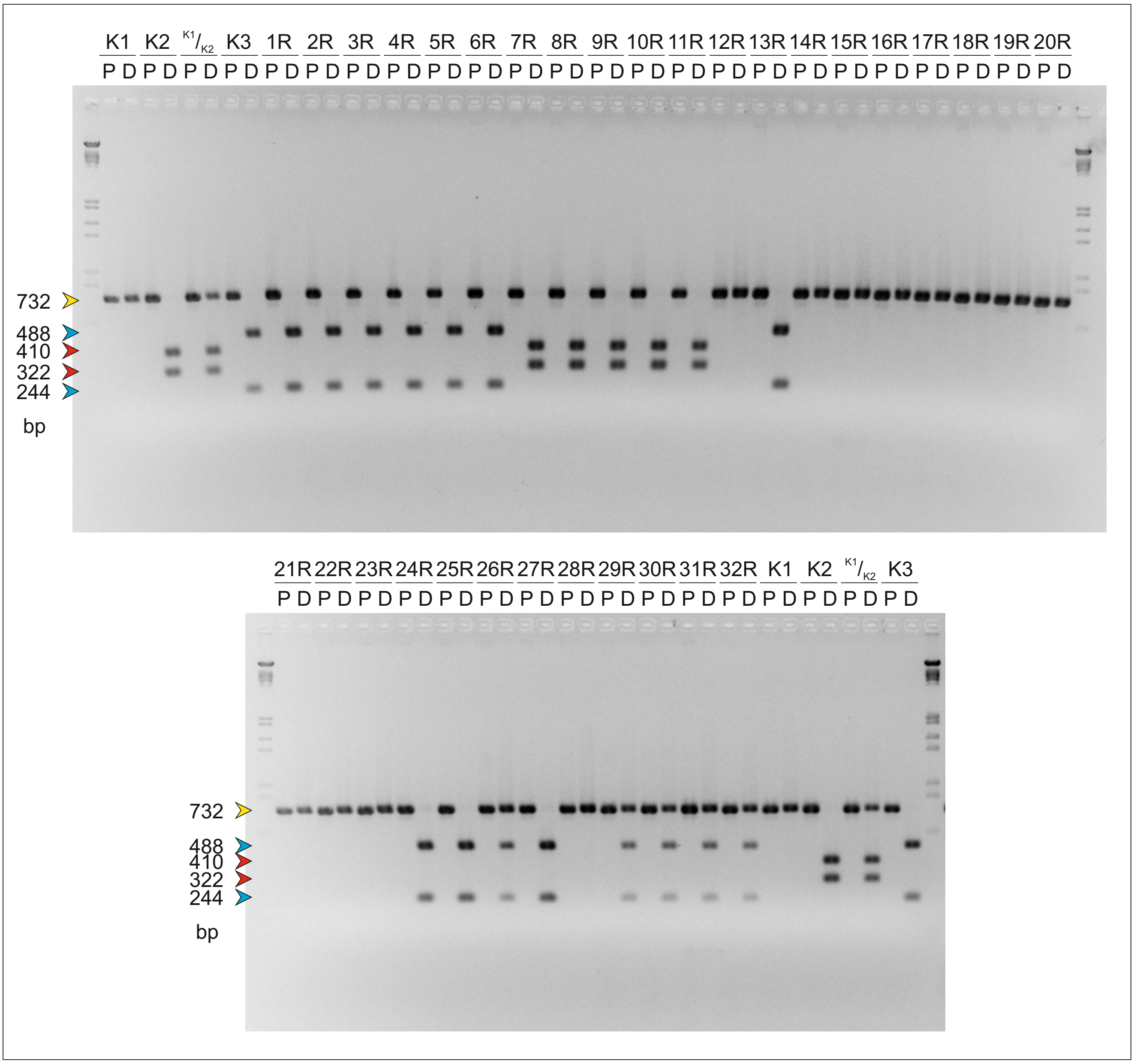

Fig. S10 The results obtained using CAPS marker analyses for the rapeseed breeding lines in 2015. For each analyzed plant, two samples (designated with letters P and D, which are explained in Fig. 2) representing two steps of the CAPS protocol were applied on the gel. The numbers above each pair of lanes refer to the description of the plants delivered by breeders in 2015. The control samples are designated as follows: K1—wild-type homozygote, K2—HOR3 type homozygote, $^{K1}/_{K2}$—mixed sample of the wild-type and HOR3 homozygotes (for the simulation of the heterozygotic genotype), and K3—HOR4 type homozygote. The arrows indicate the DNA fragments observed on the agarose gel, and their colors correspond to the colors used for the display of each mutation shown in Fig. 1

Molecular Biology Reports

Cleaved amplified polymorphic sequences (CAPS) marker for identification of two mutant alleles of the rapeseed *BnaA.FAD2* gene

Marcin Matuszczak, Stanisław Spasibionek, Katarzyna Gacek, Iwona Bartkowiak-Broda

Corresponding author: Marcin Matuszczak
Plant Breeding and Acclimatization Institute, National Research Institute, Research Division in Poznań, Poland
E-mail: marmat@nico.ihar.poznan.pl
