# Supplementary material for: Cleaved amplified polymorphic sequences (CAPS) marker for identification of two mutant alleles of the rapeseed BnaA.FAD2 gene
Source: Mol Biol Rep. 2020 Sep 26;47(10):7607–21. doi: 10.1007/s11033-020-05828-2 (PMC7588397; doi:10.1007/s11033-020-05828-2)
Supplement: Supplementary file 11 — Supplementary file11 (PDF 1799 kb) [file 11033_2020_5828_MOESM11_ESM.pdf]

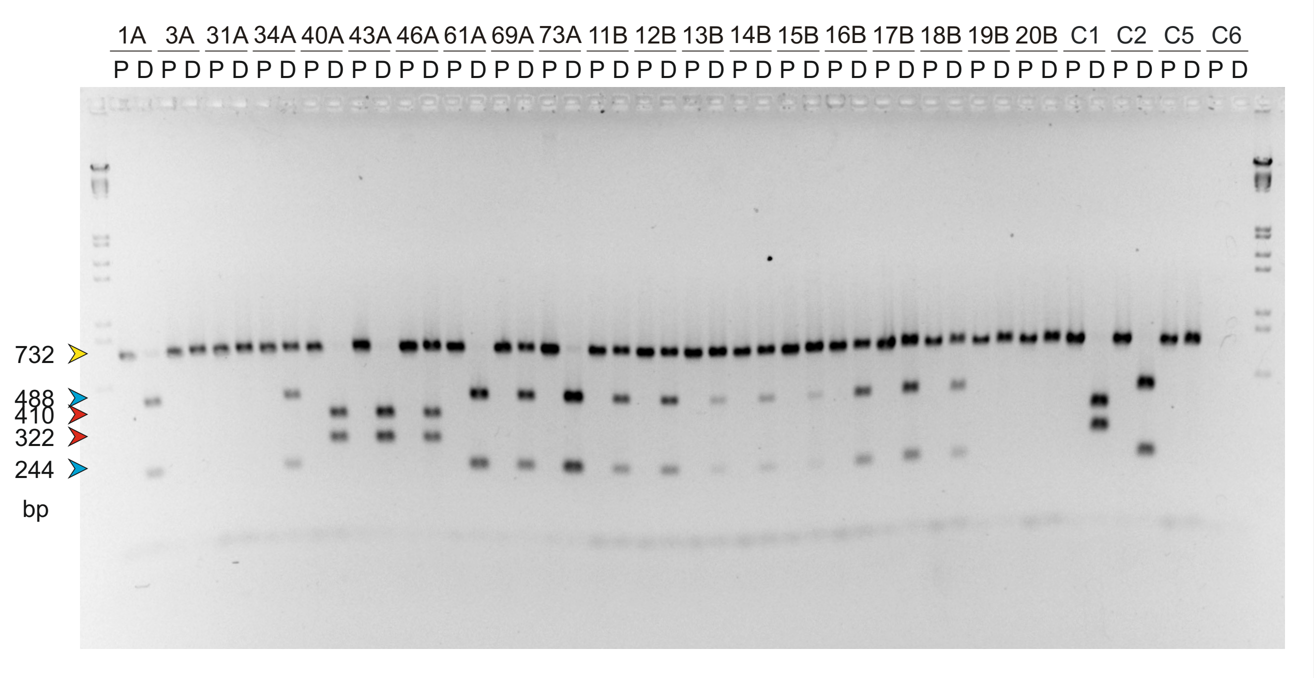

Fig. S11 An example of results obtained using CAPS marker analyses for the rapeseed breeding lines in 2016. For each analyzed plant, two samples (designated with letters P and D, which are explained in Fig. 2) representing two steps of the CAPS protocol were applied on the gel. The numbers above each pair of lanes refer to the description of the plants delivered by breeders in 2016. The control samples are designated as follows: C1—HOR3 type homozygote, C2—HOR4 type homozygote, C5—wild-type homozygote, and C6—sample without DNA. The arrows indicate the DNA fragments observed on the agarose gel, and their colors correspond to the colors used for the display of each mutation shown in Fig. 1

Molecular Biology Reports

Cleaved amplified polymorphic sequences (CAPS) marker for identification of two mutant alleles of the rapeseed *BnaA.FAD2* gene

Marcin Matuszczak, Stanisław Spasibionek, Katarzyna Gacek, Iwona Bartkowiak-Broda

Corresponding author: Marcin Matuszczak
Plant Breeding and Acclimatization Institute, National Research Institute, Research Division in Poznań, Poland
E-mail: marmat@nico.ihar.poznan.pl
