# Supplementary material for: Cleaved amplified polymorphic sequences (CAPS) marker for identification of two mutant alleles of the rapeseed BnaA.FAD2 gene
Source: Mol Biol Rep. 2020 Sep 26;47(10):7607–21. doi: 10.1007/s11033-020-05828-2 (PMC7588397; doi:10.1007/s11033-020-05828-2)
Supplement: Supplementary file 12 — Supplementary file12 (PDF 449 kb) [file 11033_2020_5828_MOESM12_ESM.pdf]

Table S1 The phenotypic characteristics of selected *B. napus* lines and cultivars compared with the results of the CAPS marker analysis for these plants. HOR3 and HOR4 type mutated alleles are named "HOR3" and "HOR4", respectively, and the wild-type alleles with no HOR3 and HOR4 type mutations are named "Wild"

Lines or cultivars	Seed yield (dt/ha)	Oil content (%)	Total of glucosinolates (μmol/g)	Fatty acid (%)			*BnaA.FAD2*[1] alleles (using CAPS marker)
				Oleic C18:1	Linoleic C18:2	Linolenic C18:3	
M681 type LL mutant line	12.0	46.6	10.7	61.0	27.5	2.7	Wild/Wild
HOR3-M10453 type HO mutant line	24.7	48.4	12.2	76.1	8.7	7.2	HOR3/HOR3
HOR4-M10464 type HO mutant line	18.6	47.7	8.7	76.6	8.8	7.4	HOR4/HOR4
PN-837 type HOLL F_9 recombinant line	14.2	43.9	13.0	76.1	12.3	2.4	HOR4/HOR4
PN-880 type HOLL F_5 recombinant line	32.0	44.6	12.7	78.3	11.2	3.2	Wild/Wild
'Monolit' cultivar	31.1	45.6	11.9	63.3	19.5	9.0	Wild/Wild

[1]Gene nomenclature follows the rules proposed by Østergaard and King [12].

Molecular Biology Reports

Cleaved amplified polymorphic sequences (CAPS) marker for identification of two mutant alleles of the rapeseed *BnaA.FAD2* gene

Marcin Matuszczak, Stanisław Spasibionek, Katarzyna Gacek, Iwona Bartkowiak-Broda

Corresponding author: Marcin Matuszczak
Plant Breeding and Acclimatization Institute, National Research Institute, Research Division in Poznań, Poland
E-mail: marmat@nico.ihar.poznan.pl
